# Whole Genome Sequencing of *Fusarium fujikuroi* Provides Insight into the Role of Secretory Proteins and Cell Wall Degrading Enzymes in Causing Bakanae Disease of Rice

**DOI:** 10.3389/fpls.2017.02013

**Published:** 2017-11-27

**Authors:** Bishnu M. Bashyal, Kirti Rawat, Sapna Sharma, Deepika Kulshreshtha, S. Gopala Krishnan, Ashok K. Singh, Himanshu Dubey, Amolkumar U. Solanke, T. R. Sharma, Rashmi Aggarwal

**Affiliations:** ^1^Division of Plant Pathology, ICAR-Indian Agricultural Research Institute, New Delhi, India; ^2^Division of Genetics, ICAR-Indian Agricultural Research Institute, New Delhi, India; ^3^ICAR-National Research Centre on Plant Biotechnology, New Delhi, India

**Keywords:** *Fusarium fujikuroi*, bakanae, whole genome sequencing, secretome, comparative genomics

## Abstract

*Fusarium fujikuroi* causing bakanae disease has emerged as one of the major pathogen of rice across the world. The study aims to comparative genomic analysis of *Fusarium fujikuroi* isolates and identification of the secretary proteins of the fungus involved in rice pathogenesis. In the present study, *F. fujikuroi* isolate “F250” was sequenced with an assembly size of 42.47 Mb providing coverage of 96.89% on reference IMI58289 genome. A total of 13,603 protein-coding genes were predicted from genome assembly. The average gene density in the *F. fujikuroi* genome was 315.10 genes per Mb with an average gene length of 1.67 kb. Additionally, 134,374 single nucleotide polymorphisms (SNPs) are identified against IMI58289 isolate, with an average SNP density of 3.11 per kb of genome. Repetitive elements represent approximately 270,550 bp, which is 0.63% of the total genome. In total, 3,109 simple sequence repeats (SSRs), including 302 compound SSRs are identified in the 8,656 scaffolds. Comparative analysis of the isolates of *F. fujikuroi* revealed that they shared a total of 12,240 common clusters with F250 showing higher similarity with IMI58289. A total of 1,194 secretory proteins were identified in its genome among which there were 356 genes encoding carbohydrate active enzymes (CAZymes) capable for degradation of complex polysaccharides. Out of them glycoside hydrolase (GH) families were most prevalent (41%) followed by carbohydrate esterase (CE). Out of them CE8 (4 genes), PL1 (10 genes), PL3 (5 genes), and GH28 (8 genes) were prominent plant cell wall degrading enzymes families in F250 secretome. Besides this, 585 genes essential for the pathogen–host interactions were also identified. Selected genes were validated through quantitative real-time PCR analyses in resistant and susceptible genotypes of rice at different days of inoculation. The data offers a better understanding of *F. fujikuroi* genome and will help us enhance our knowledge on *Fusarium fujikuroi*–rice interactions.

## Introduction

Bakanae is an important fungal disease of rice posing threat to rice production all over the world. The disease incidence of bakanae on rice has increased at an alarming rate taking its toll in diverse rice growing regions such as India, Japan, Taiwan, and Thailand (Kini et al., 2002; Saremi, 2005; Bashyal and Aggarwal, 2013; Gupta et al., 2015; Bashyal et al., 2016a; Fiyaz et al., 2016). Apart from causing significant yield losses (Ou, 1985), the bakanae pathogen also affects seed quality (Bashyal et al., 2014, 2016b; Fiyaz et al., 2014).

The fungus associated with the bakanae disease is *Fusarium fujikuroi* (Nirenberg), [teleomorph: *Gibberella fujikuroi* (Sawada) Ito]. The fungus causes chlorotic symptoms leading to reduced seed germination and abnormally tall and weak plants thereby resulting in plant death. Infected seedlings are stunted and exhibit rotting symptoms with powdery growth of conidiophores on the lower parts (Sun and Snyder, 1981; Webster and Gunnell, 1992). The incidence of bakanae disease has increased steadily particularly on Basmati rice cultivars in north-west India especially in Punjab, Haryana, and Uttar Pradesh (Gupta et al., 2015; Bashyal et al., 2016a).

Rapid advances in sequencing technologies have enabled development of reference genomes in different organisms and identification of genome-scale variations (Subbaiyan et al., 2012). Over the past decade, efforts have been made for sequencing multiple *Fusarium* genomes, which in conjunction with whole genome shot gun sequencing has helped in identification of genome-wide variations and understanding virulence mechanisms and pathways (King et al., 2015). Till now five genomes of *F. fujikuroi* are published from different countries (Jeong et al., 2013; Wiemann et al., 2013; Chiara et al., 2015). However, most of the studies on *F. fujikuroi* were focused on species – specific and isolate specific differences in the composition and expression of genes involved in secondary metabolite production. Zhao et al. (2013) reported the cell wall degrading enzymes like cellulases, β-glycosidases and accessory enzymes from the maize pathogen *F. verticillioides*. The rice GHs are classified into 34 families encoding 437 genes (Sharma et al., 2013). However, none of the studies reported secretory proteins and cell wall degrading enzymes in the genome of *F. fujikuroi.*

Considering the above facts, the present study was carried out to understand its genome and its function through whole genome sequencing and comparative genomic analysis of *F. fujikuroi*. Whole genome of *F. fujikuroi* isolate “F250” was sequenced and comparative genomic analyses was performed with five other recently published *F. fujikuroi* genome sequences (Jeong et al., 2013; Wiemann et al., 2013; Chiara et al., 2015). Further, secretome analysis of the sequences of “F250” was also conducted to identify pathogenesis/virulence related genes and CAZymes (related to cell wall degrading enzymes) which have been validated in resistant and susceptible genotypes of rice. Overall, the present study was aimed at providing new insights into the genome of *F. fujikuroi* as well as to develop better understanding of *F. fujikuroi*–rice plant interaction which can help in developing effective management strategies for bakanae disease.

## Materials and Methods

### Culture Conditions and DNA Isolation

The virulent *F. fujikuroi* isolate “F250” (GenBank Accession no. KM50526) isolated from symptomatic bakanae infected rice plant in Hisar, Haryana (India) was used for whole genome sequencing (Bashyal et al., 2016a). Genomic DNA was extracted by following the protocol of Murray and Thompson (1980) with slight modification. The cultures were grown in potato dextrose broth (PDB; pH 5.5) for 7 days at 25 ± 1°C in a shaker incubator. Filtered mycelium (5 g) was ground to a fine powder in liquid nitrogen, transferred to 10 ml of CTAB buffer (0.1 M Tris, 1.5 M NaCl, 0.01 M EDTA and 2% CTAB) in a 50 ml tube and kept at 65°C for 1 h with occasional stirring. An equal volume of chloroform: isoamyl alcohol (24:1) was added to all the tubes, followed by centrifugation at 10,000 rpm for 20 min. The upper aqueous phase was pipetted out in a fresh tube mixed with 0.1 volume of sodium acetate (3M) and 0.6 volume of chilled isopropanol and centrifuged again at 10,000 rpm for 10 min at 4°C. The pellet was washed with 70% ethanol, dried at room temperature and finally dissolved in TE (10 mM Tris-HCl, pH 8.0; 1 mM EDTA) for further use. Quality was checked on 1% agarose gel (loaded 5 μl) for the single intact band. The gel was run at 110 V for 30 min. One microliter of each sample was loaded in Nanodrop 2000 for determining A260/280 ratio, and 1 μl of each sample was used for determining concentration using Qubit^®^ 2.0 Fluorometer (Thermo Fischer Scientific, Life Technologies). For the determination of mating type of the isolate MAT-1 primers GFmat1a (5′-GTTCATCAAAGGGCAAGCG-3′) and GFmat1b (5′-TAAGCGCCCTCTTAACGCCTTC-3′) were used and the primer pairs Gfmat2c (5′-AGCGTCATTATTCGATCAAG-3′) and Gfmat2d (5′-CTACGTTGAGAGCTGTACAG-3′) were used for the MAT-2 region as described by Steenkamp et al. (2000).

### Genome Sequencing and Assembly

The library was prepared from the *Fusarium fujikuroi* sample “F250” using TruSeq Nano DNA HT Library Sample Preparation Kit. The mean size of the library was 685 bp. Whole genome sequencing was performed using 2 × 150 bp chemistry on the Illumina NextSeq platform. Filtered high quality sequence reads were assembled through “GS Reference Mapper” version 2.3 (Roeche Inc., Germany) with default parameters (minimum read length = 20 bp, minimum overlap length = 40 bp, minimum overlap identity = 90%, alignment identity score = 2 and all contig threshold = 100) using *F. fujikuroi* isolate IMI58289 as reference genome. MUMmer 4.0 software (Kurtz et al., 2004) was used to align total assembled RML-29 contigs on the reference genome. Furthermore, the *de novo* assembly of the unassembled reads as well as the alignment of the raw reads was performed using CLC Genomics Workbench 7.0 with default parameters (minimum contig = 100 bp, 23 K-mer, similarity fraction = 80% and length fraction = 50%).

### Gene Prediction

Protein-coding genes in the *F. fujikuroi* isolate “F250” masked genome were predicted using FGENESH 3.1.2 (MolQuest 2.2). Inhouse developed PEARL scripts were used to parse the FGENESH output and extract sequences. FGENESH was trained with *Fusarium graminearum* that predicted a total of 14,302 protein coding genes. Out of them, 629 genes were discarded due to less than 100 nucleotide and 13,603 were taken for the further study.

### Genome Annotation

For functional annotation of *F. fujikuroi* predicted genes, BLASTX search against NCBI non-redundant database was performed with cut-off E-values of ≤1e-5 and identity ≥40%. Gene ontology (GO) analysis was carried out using BLAST2GO (Blast2GO, 2016).

### Identification of Repetitive Elements and Simple Sequence Repeats (SSRs)

The repetitive elements belonging to various classes including long terminal repeats (LTRs), non-LTRs, DNA transposons, etc., were identified using RepeatMasker version open-4.0.6, sensitive mode run with rmblastn version 2.2.27+ RepBase Update 20150807, RM database version 20150807 was used to identify repetitive families of repetitive sequences in the *F. fujikuroi* genome. Mining of SSRs was done using MISA software and categorized using standard parameters (MISA, 2016).

### Detection of Single Nucleotide Polymorphism (SNP)

Single nucleotide polymorphisms were detected using Sequence Alignment/Map tools (SAM tools) software package at 10× coverage with the quality value of Phred score ≥ 20. The SAM files generated by BWA was converted to bam file and processed by mpileup utility of SAM tool to generate a pile-up of read bases using the alignments to the reference sequence (IMI 58289) for the prediction of SNPs. Additionally, SNP detection by CLC Workbench 7.0 was also performed (parameters, Ploidy = 2, coverage 10–100,000, variant frequency ≥ 35%. The annotation of the SNPs (SAM tools) was performed using SnpEff software by default parameters (Cingolani et al., 2012).

### Secretome Prediction and Analysis

A set of 13,603 protein from *F. fujikuroi* was analyzed in SignalP v4.1 (SignalP, 2016), TargetP version 1.1 (TargetP, 2016) and Phobius (Phobius, 2016) for the prediction of the secretory signal peptide. Initially, proteins (>30 amino acids) with a SignalP D-Score = Y and a cut off value, 0.45 for 0 Tm/0.50 for 0.50 Tm and TargetPLoc = S were combined. After this, the protein sets were scrutinized for the presence of transmembrane domain using TMHMM (v2.0; TMHMM, 2016). Peptides with 0 or 1 transmembrane regions were retained and transmembrane region located in less than 10 amino acids in mature peptide from predicted cleavage site as well as proteins with highly probable GPI (glycosylphosphatidyl inositol) anchor predicted by PredGPI (2017) were taken for further analysis. WoLFPSORT analysis was performed using “run WoLFPSORT v. 0.2” (WoLFPSORT, 2017). The predicted secretome was functionally annotated by assigning GO terms using BLAST2GO and BLASTP analysis with *e* < 1*e*- 05 cut-off against PHIbase: the pathogen–host interaction gene database (PHI, 2017) was performed to predict potential pathogenicity genes associated with loss of pathogenicity, reduced virulence, lethal, etc. (Winnenburg et al., 2006). The CAZymes Analysis Toolkit (CAT) was used to detect carbohydrate active enzymes (CAZymes) based on the CAZy database (Cazy, 2016). An annotation method based on association rules between CAZy families and Pfam domains was used with an *E*-value threshold of 0.01, a bitscore threshold of 55 and rule support level 40.

### Comparative Analysis of Orthologous Gene Families

The orthologous groups among different isolates of *F. fujikuroi* were identified with the help of Orthovenn of UC Davis, for which fungi dataset was selected and protein sequences of six different isolates (**Table 1**) including F250 was uploaded with default *E*-value of 1*e* - 5 and inflation value of 1.5.

**Table 1:** List of *F. fujikuroi* isolates used in the study including “F250” which was sequenced in this study.

Isolate	Pathogen	Origin	Reference
F250	*F. fujikuroi*	India	Fiyaz et al., 2014, 2016; Bashyal et al., 2016a
IMI58289	*F. fujikuroi*	Taiwan, rice	Wiemann et al., 2013
FGSC 8932	*F. fujikuroi*	Taiwan, rice	Chiara et al., 2015
KSU 3368	*F. fujikuroi*	Thailand, rice (1990)	Chiara et al., 2015
KSU X-10626	*F. fujikuroi*	Konza Prairie (United States), *Schizachyrium scoparium* (1997)	Chiara et al., 2015
B14	*F. fujikuroi*	South Korea, rice	Jeong et al., 2013
ITEM 7600	*F. verticillioides*	California, maize	Leslie and Dickman, 1991

### Phylogenetic Analysis of *F. fujikuroi* Genomes

Progressive Mauve version 20150226 build 1,045 with default parameters was used to perform phylogenetic analysis based on whole-genome alignment. Six isolates of *F. fujikuroi* namely F250 IMI58289, FGSC 8932, KSU 3368, KSU X-10626, B14 and one isolate of *F. verticillioides* ITEM 7600 (**Table 1**) were taken for the analysis. Comparative analysis was performed on all seven genomes by studying the neighbor joining tree produced as described by Saitou and Nei (1987).

### Quantitative Real-Time PCR

#### Inoculation of Pathogen and Sample Collection

For the validation of selected genes of secretome (genes present in PHI database and cell wall degrading enzymes) the highly susceptible rice cultivar Rasi and comparative resistant genotype C101A51 were selected (Fiyaz et al., 2014). *F. fujikuroi* isolate F250 was subcultured on sterile sorghum grains and incubated at 25°C. After 15 days the spore suspension was prepared with sterile distilled water, filtered through two layers of sterile muslin cloth and brought to a final concentration of 10^6^ spores ml^-1^. For the inoculation, seeds obtained from Division of Genetics, Indian Agricultural Research Institute, New Delhi, were surface-disinfected by immersion in 70% ethanol for 1 min, transferred to 1% sodium hypochlorite for 3 min and rinsed three times in sterile distilled water consecutively. The seeds were then dried in Petri dishes containing sterile filter paper. Rice seeds of resistant and susceptible genotypes were kept in inoculum suspension for 18 h at room temperature (25°C). Control seeds were soaked in sterile water. Inoculated and control seeds were sown in pots as 10 seeds per pot and 20 pots per genotype containing autoclaved mixture of soil and sand in the ratio of 3:1. The green house temperature was maintained at 30–35°C during the day and 16–18°C during the night. As disease started appearing after 7 days of inoculation, and maximum disease appears after 30 days of inoculation or 3 weeks after germination (Bashyal et al., 2016a; Matic et al., 2016) 7, 10, and 30 days were selected for the gene expression analysis.

#### Quantitative Real-Time PCR Analysis for the Selected Genes

The rice seedlings of susceptible and resistant genotypes were carefully uprooted, at different time interval of inoculation, washed in running tap water and homogenized with liquid nitrogen in a pre-chilled mortar and pestle. Total RNA was isolated using Tri-reagent (Invitrogen) according to the manufacturer’s protocol from the powdered samples (100 mg). For the reverse transcriptase-polymerase chain reaction (RT-PCR), 2 mg of RNA was treated with RNase-free DNase (Genetix) for 30 min at 37°C followed by DNase stop solution for 10 min at 65°C for DNase inactivation. The treated RNA (2 μg) was added to oligo (dT) 12–18 primer, and the sample (20 μl) was briefly denatured at 65°C for 10 min and chilled on ice for 2 min. Reverse transcription was carried out following the protocol supplied with MuMLv (Fermentas) at 42°C for 1 h. For quantitative PCR study individual genes involved in different cell wall degrading enzyme families and host–pathogen interactions were randomly selected based on their function of blast search (BLAST, 2016) against the GenBank database^1^ as described in Supplementary Table 2. The primer sets for the randomly selected genes related to cell wall degrading enzymes and genes involved in pathogen–host interactions (PHI-database) was designed using the IDT oligo analyzer^2^ at default settings (Supplementary Table 3). An *in silico* test for primer specificity was conducted by running the primer sequence against the non-redundant GenBank data^1^ with parameters set for the identification of short, nearly exact matches (Supplementary Table 3). Quantification of gene expression was performed through MJ miniopticon-48 wells real time PCR detection system (Bio-Rad labs, Inc.). The PCR mixture contained 100 ng of cDNA template, 10 μl of 2X dynamo color flash SYBR green mix dye (Thermo Scientific, United States), 100 nM forward and reverse primer in a final volume of 20 μl. Thermal cycling conditions were as follows: 95°C for 10 min followed by 39 cycles of 95°C for 15 s and 53°C for 30 s and a melt curve from 65°C to 95°C. Melt curve and threshold data was observed during each cycle. Relative gene expression by qPCR was performed using *Actin* as the reference gene for normalization of expression of target genes (Huggett et al., 2005). PCRs with no template controls (NTC) were also performed for each primer pair. The specificity of amplicons was verified by melting curve analysis (60–95°C) after 40 cycles. The fold change in the target gene, relative to the expression of *Actin* was calculated using 2^-ΔΔ*C*_T_^ method (Livak and Schmittgen, 2001). The mean and SE (±), were then determined for each sample at different time interval. Three biological replicates for each sample were used for real-time PCR analysis and three technical replicates were analyzed for each biological replicate.

## Results

### Genome Sequencing, Assembly, and Annotation

**FIGURE 1:**
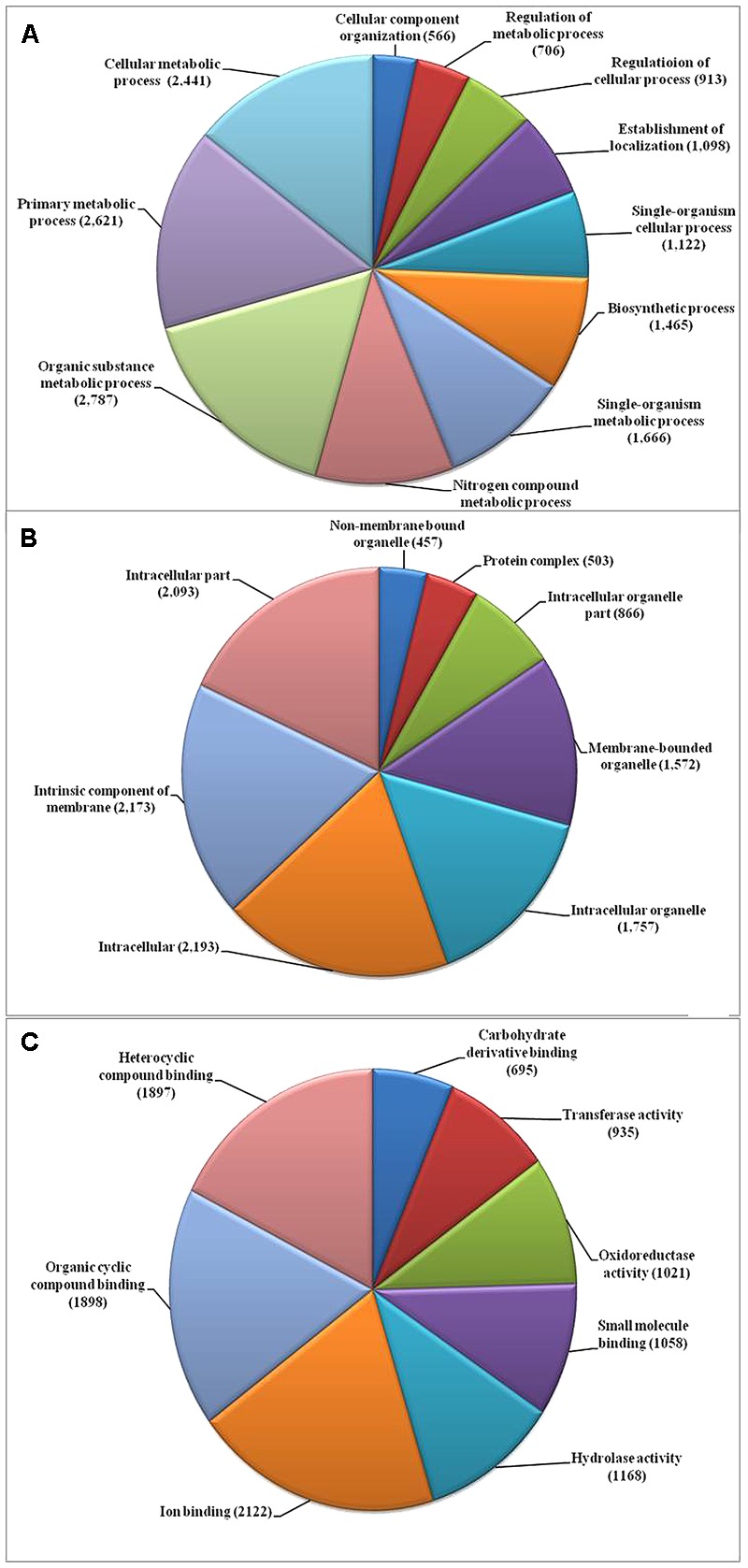
Gene ontology (GO) based functional annotation of genes present in the *Fusarium fujikuroi* genome. **(A)** Biological process domains; **(B)** cellular process domains; and **(C)** molecular function domain.

We used the highly virulent Indian isolate (F250) of *F. fujikuroi* (GenBank Accession no. KM50526; Supplementary Figure 1) for sequencing. This isolate was identified as mating type 2 (MAT-2) using MAT locus-specific primers. Out of 6,30,22,29,117 raw reads, 43,176,754 were mapped to the reference genome. Filtered high quality paired-end reads were assembled using ABySS5, resulting in a total assembly size of 42.47 Mb with coverage of 96.89% of reference genome. Overall coverage of 146X was obtained as estimated by *k*-mer analysis of read count versus *k*-mer coverage and consisted of 3,889 scaffolds with N50 scaffold size of approximately 138.72 kb (**Table 2**). In total, 13,603 protein-coding genes were predicted from genome assembly using FGENESH. The average gene density in the *F. fujikuroi* genome was 315.10 genes per Mb (**Table 2**). The average gene length was 1.67 (kb) and consisted of an average of 0.52 (kb) of coding region and 0.083 (kb) of non-coding region. The overall GC content of the *F. fujikuroi* “F250” genome was estimated to be 47.64%. BLASTP resulted in the annotation of 13,565 CDS for the F250 isolate. The majority of the hits were found to be against the *Fusarium fujikuroi* followed by *F. fujikuroi* IMI58289 isolate (Supplementary Figure 2). GO mapping was carried out in order to retrieve GO terms for all the BLASTP functionally annotated CDS. Out of them 5,287 genes belonged to biological process, 4,218 genes belonged to cellular component, and 5,456 genes were assigned to molecular function (**Figure 1**). Additionally, a total of 134,374 SNPs were identified in comparison with the reference genome of IMI58289 isolate. The average SNP density was 3.11 per Kb of genome.

**Table 2:** Genome features of *Fusarium fujikuroi* isolate “F250”.

Data type	F250
Size (Mb)	42.47 (Mb)
Coverage	96.89%
% (G + C) content	47.64%
% Repeat	0.63%
Protein-coding genes	13,603
Average gene length (bp)	1.67 (kb)
Gene density (number of genes per Mb)	31.51 gene/100 kb
Average exons per gene	2.90 exon/gene
Average exon length (bp)	0.52 (kb)
Average introns per gene	1.91 intron/gene
Average intron length (bp)	0.083 (kb)
Secreted proteins	1,691
SNPs	134,374

### Repetitive Elements and Simple Sequence Repeats (SSRs)

**FIGURE 2:**
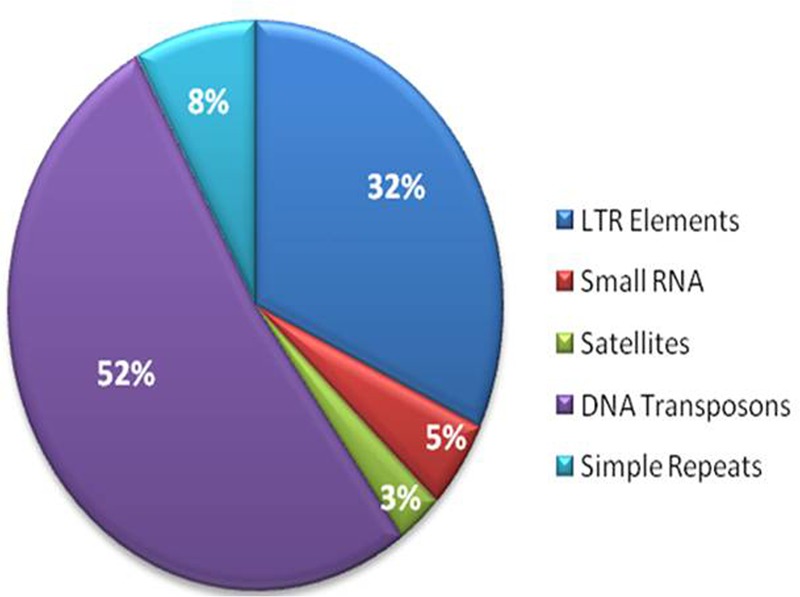
Percentage distribution of different types of repetitive elements in the genome of *Fusarium fujikuroi* isolate “F250”. These repetitive elements are approximately 270,550 bp, which represents 0.63% of the total genome.

The repetitive elements in the *F. fujikuroi* “F250” genome were estimated to be approximately 270,550 bp, which represent 0.63% of the total genome. Out of them, 52% of the transposable elements (TEs) were DNA transposon, Tc1-IS630-Pogo covering 52% of repetitive elements (**Table 3** and **Figure 2**) followed by LTR elements (32%). A high-throughput SSR search to identify mono- to hexanucleotide SSR motifs in the *F. fujikuroi* genome was performed. In total, 3,109 SSRs, including 302 compound SSRs were identified (**Table 4**). Of all compound SSRs, 100 interrupted SSRs (C) constituted 33.12% of the compound SSRs. In contrast, 202 uninterrupted SSRs (C^∗^) were found. The dinucleotide AT repeats were found to be the predominant, followed by trinucleotide ATC/ATG repeats.

**Table 3:** Repetitive elements identified in the *F. fujikuroi* isolate “F250”.

Repetitive	Number of	Length of	Percentage
elements	elements^∗^	sequence	
LTR elements	341	86,184 bp	0.20%
Ty1/Copia	59	4,912 bp	0.01%
Gypsy/DIRS1	282	81,272 bp	0.19%
DNA transposons	653	142,552 bp	0.33%
Tc1-IS630-Pogo	620	140,283 bp	0.32%
Others	12	779 bp	0.01%
Unclassified	4	197 bp	0.00
Retroelements	358	87,486 bp	0.20%
Total interspersed repeats	–	230,235 bp	0.53%
Small RNA	50	12,494 bp	0.03%
Satellites:	65	7,068 bp	0.02%
Simple repeats	252 bp	20,095	0.05%
Low complexity	4	658 bp	0.00%

**Table 4:** Number of SSRs identified in the *F. fujikuroi* isolate “F250” genome and their distribution to different repeat type classes.

Unit size	Number of SSRs
1 (1/10)	1,592
2 (2/6)	566
3 (3/5)	613
4 (4/5)	201
5 (5/5)	100
6 (6/5)	37
Total	3,109

### Prediction and Analysis of *F. fujikuroi* Secretome

**FIGURE 3:**
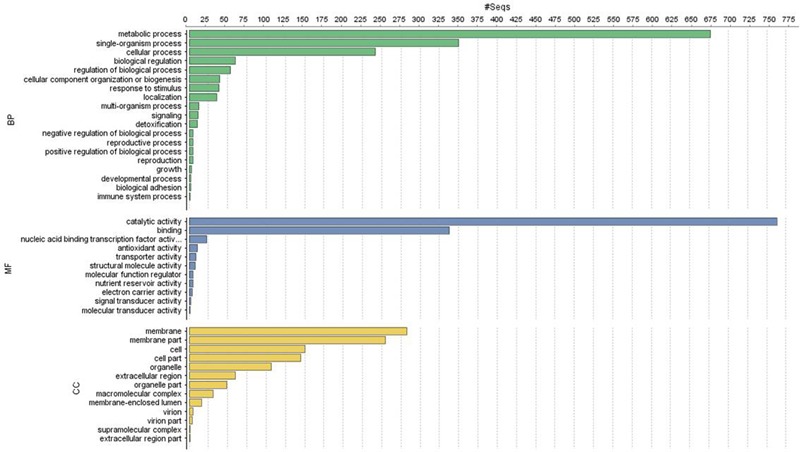
Functional annotation of the *Fusarium fujikuroi* secretome showing top 20 hits of different category. BP, biological process; MF, molecular function; CC, cellular component.

**FIGURE 4:**
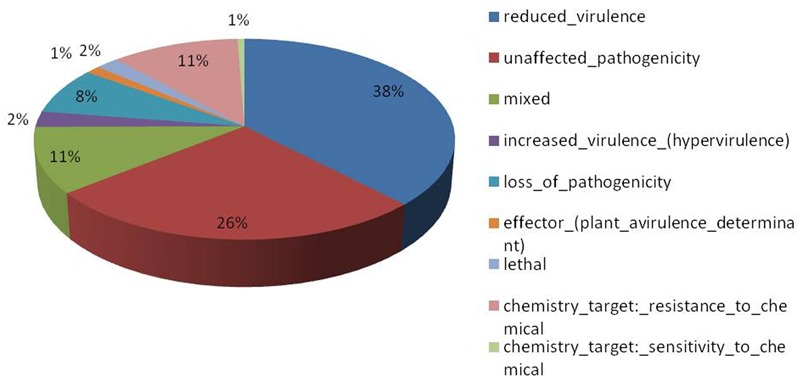
Summary of different phenotypic categories of orthologs of *Fusarium fujikuroi* secretome genes in pathogen–host interactions (PHI-base) database.

**FIGURE 5:**
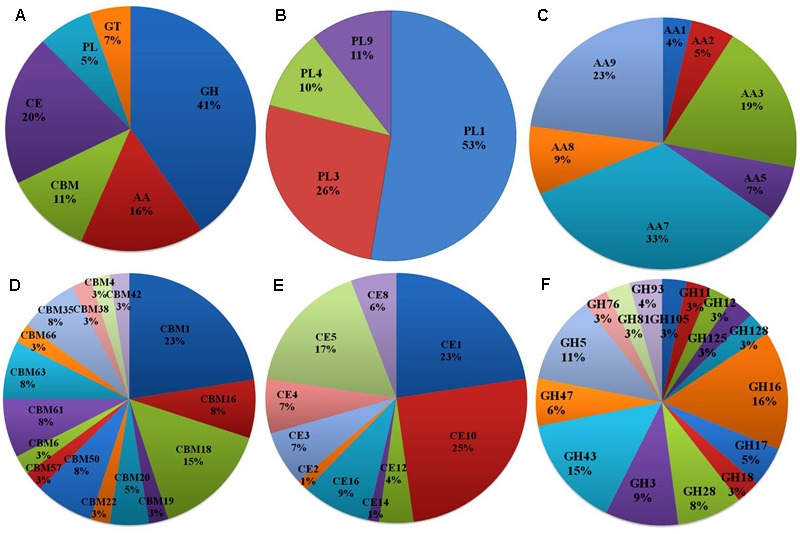
CAZymes identified in the secretome of *F. fujikuroi*; **(A)** Summary of the six CAZyme categories: carbohydrate-binding modules (CBMs), carbohydrate esterases (CEs), glycoside hydrolases (GHs), glycosyl transferases (GTs), polysaccharide lyases (PLs), and auxiliary activities (AAs). **(B)** Distinct summaries of the CAZyme PLs. **(C)** Distinct summaries of each of the CAZyme and auxiliary activities. **(D)** Distinct summaries of each of the CAZyme GHs. **(E)** Distinct summaries of each of the CAZyme carbohydrate-binding modules (CBMs). **(F)** Distinct summaries of each of the CAZyme GHs.

Of the 13,603 proteins, a total of 1,207 proteins could be annotated as classical secretory proteins by SignalP version 4.1, while TargetP version 1.1 classified 2,265 proteins as secretory proteins. After merging the filtered sets (SignalP and TargetP) and the removal of duplicate segments, a total of 2,265 proteins were scanned using TMHMM software. A total of 1,691 sequences were predicted as secretory proteins, after the removal of 574 transmembrane proteins from the protein data set. Secretory proteins predicted in the previous step were further screened using WoLFPSORT version 3, resulting in 1,194 proteins. For analyzing predicted *F. fujikuroi* secretome, GO terms were assigned to 985 putative secretory proteins in three GO categories namely, molecular function (736), biological process (670), and cellular component (280) (**Figure 3**). Under biological process, categories such as single-organism process, cellular metabolic process, cellular process, biological regulation, regulation of biological process, cellular component organization or biogenesis, response to stimulus and localization were highly represented. Within molecular function ontology, proteins associated with binding, antioxidant activity, transporter activity, structural molecule activity were most abundant. In the cellular component category, proteins for membrane part, cell and cell part, organelle part, macromolecular complex were highly abundant. Out of 1,194 secretory proteins, 585 proteins have shown matches with PHI-database in different categories. Out of them 38% proteins were related to reduced virulence, 26% proteins are related to unaffected pathogenicity and 11% proteins are of mixed nature (**Figure 4**). Using CAZy database and performing HMMER scan based on the profile compound in DBscan release 2.0, presence of 5% polysaccharide lyases (PLs), 7% glycosyl transferases (GTs), 16% auxiliary activities (AAs), 11% carbohydrate binding modules (CBMs), 20% carbohydrate esterases (CEs) and 41% glycosyl hydrolases (GHs) were predicted in the *F. fujikuroi* “F250” secretome. Of the six CAZy classes further analysis was performed for the cell wall degrading enzymes families. Out of 51 GH families identified, 16 GH families showed the presence of three or more than three genes. Maximum genes (15) were present for the family GH16 followed by GH43 (14 genes), GH5 (11 genes), and GH3 (9 genes). Ten CE families were present in the secretome of *F. fujikuroi*. Maximum no. of genes were identified for CE10 (18 genes) followed by CE1 (16 genes), CE5 (12 genes), and CE16 (6 genes). Further, four PL families PL1 (10 genes), PL3 (5 genes), PL4 (2 genes), and PL9 (2 genes) were also predicted. Further, classes with indirect roles in degrading carbohydrates like AA (7 families), CBM (16 families), and GT (25 families) were also identified. Out of them CBM-01, AA7, GT-34 families were most abundant (**Figure 5** and Supplementary Table 1). *F. fujikuroi* secretome also contained distinct oxidoreductases, transferases, hydrolases and lyases. Therefore, these analysis suggested that the secretome of *F. fujikuroi* consists of proteins of diverse nature, which might function in facilitating proper colonization of the fungus, degradation of the host plant matter to acquire nutrients and inactivation of the host defenses. Secretome of the *F. fujikuroi* isolate “F250” is closely related to *F. fujikuroi* isolate IMI58289 followed by different species of *F. oxysporum* (Supplementary Figure 3).

### Comparison of Orthologous Genes between Different *F. fujikuroi* Isolates

**FIGURE 6:**
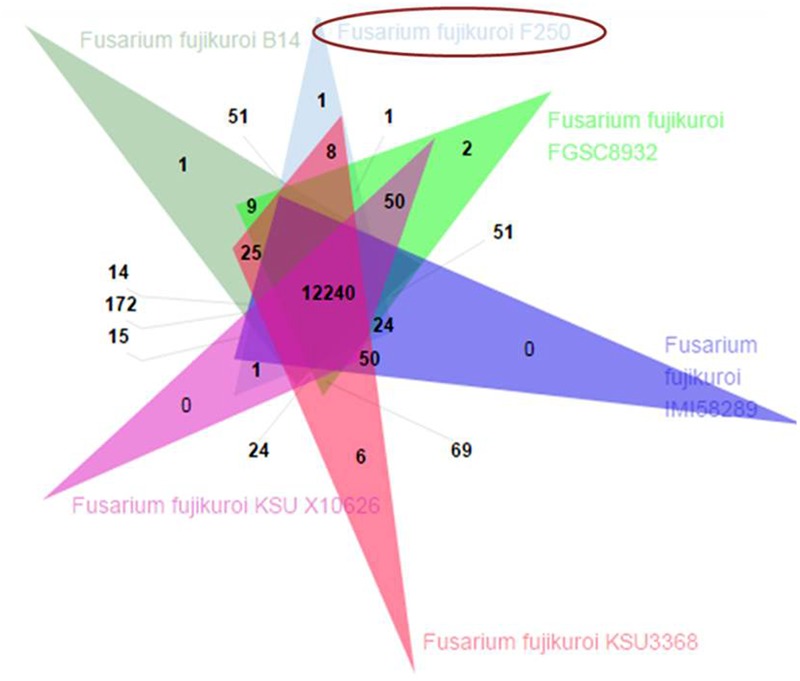
Venn diagram showing unique and shared orthologous gene families between the *F. fujikuroi* isolates. The orthologous gene families among the *Fusarium fujikuroi* isolates B14, FGSC 8932, IMI58289, KSU3368, KSU X10626, and F250 were identified using OrthoVenn. Comparison of the six isolates revealed 12,240 gene families are common in all the isolates.

The predicted proteome of *F. fujikuroi* (F250) was compared with other isolates of *F. fujikuroi* namely IMI58289, FGSC 8932, KSU 3368, KSU X-10626, and B14 (**Figure 6**). Results indicated that these isolates form 15,316 clusters, out of which the isolates of *F. fujikuroi* shared a total of 12,240 common clusters. These common clusters were subjected to functional annotation. A total of 152 GO terms were assigned for biological process, 56 GO term assigned for molecular function and 99 GO terms were assigned for the cellular functions. A total of 24 common clusters were present between the isolates, F250 and IMI58289; 8 common clusters between F250 and KSU3368; 1 cluster between F250 and FGSC8932; and 51 common clusters between F250 and B14. Two unique clusters were present in FGSC8932 and 1 unique cluster was present in isolate B14. While six unique clusters were observed in KSU3368.

### Phylogenetic Analysis

**FIGURE 7:**
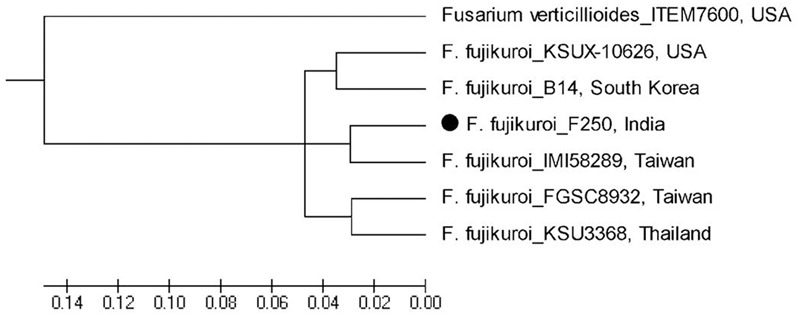
Whole genome phylogenetic analysis of *Fusarium fujikuroi* sequences after available. *F. verticillioides* was used as outgroup.

Based on whole genome phylogenetic analysis, all the isolates of *F. fujikuroi* grouped together in a single major cluster showing 96–100% similarity to each other. Further, phylogenetic analysis of *F. fujikuroi* isolates indicated that *F. fujikuroi* isolate “F250” is closer to “IMI58289” as they were grouped together in subcluster 2. Isolates KSU X-10626 and B14 were grouped together in subcluster I and isolates FGSC8932 and KSU3368 were grouped in subcluster III. *F. verticillioides* isolate “ITEM 7600” grouped separately as an outgroup (**Figure 7**).

### Quantitative Real-Time PCR

**FIGURE 8:**
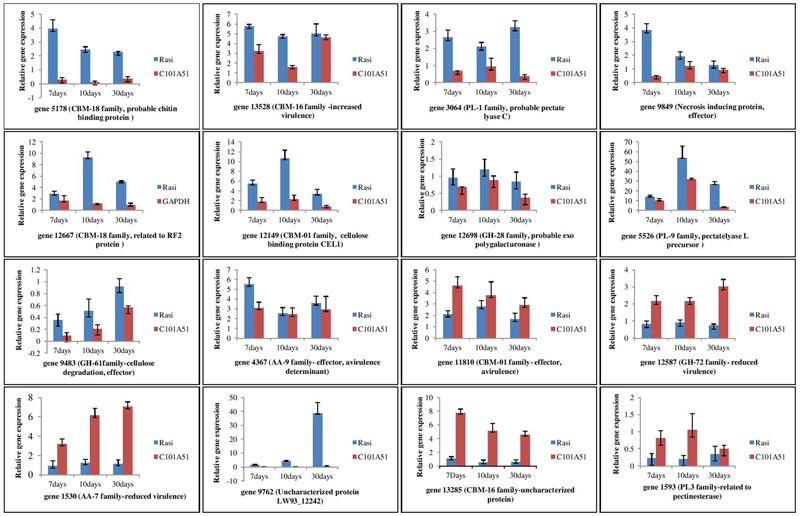
qPCR based analysis of *F. fujikuroi* secretome genes at different time of inoculation in resistant rice genotype, C101A51 and susceptible genotype, “Rasi”. Mean values ± standard error of triplicate data from three biological replicates are shown.

Differences were observed for the gene expression at different time interval of inoculation in resistance and susceptible rice genotypes (**Figure 8**). These genes are generally categorized into five groups based on their expression profiles during infection. The first group includes genes 5,178 (probable chitin binding protein/CBM-18 family), 13,528 (related to *O*-methylsterigmatocystin oxidoreductase/CBM-16 family), 3,064 (probable pectate lyase C/PL-1 family), 9,849 (uncharacterized protein FFUJ_12628/necrosis inducing protein), 4,367 (related to cellulose binding protein CEL1), and 1,593 (related to pectinesterase/PL-3 family) where maximum expression was observed after 7 days of inoculation. Second group consisted of genes 12,667 (related to RF2 protein/CBM-18 family), 12,149 (cellulose binding protein CEL1/CE-1 family), 12,698 (probable exopolygalacturonase/GH-28 family), and 5,526 (pectate lyase L precursor/PL-9 family) where maximum expression was observed after 10 days of pathogen inoculation. Three genes 9,483 (uncharacterized protein LW93_4415/Glycosyl hydrolase family 61), 12,587 [probable beta (1-3) glucanosyltransferase/GH-72 family], and 1,530 (6-hydroxy-d-nicotine oxidase/AA-7 family) comprised of third group which were up-regulated and gradually increased during infection and maximum expression is observed after 30 days of inoculation. Gene 9762 (Uncharacterized protein LW93_12242/Cytochrome P450) is expressed in susceptible genotype only categorized in fourth group. Genes 13285 (uncharacterized protein FFUJ_06869/CBM-16 family) and 1593 (related to pectinesterase/PL-3 family) constituted fifth group where expression level was almost constant at different time interval of inoculation. The results demonstrated that all 16 secretory protein-encoding genes were differentially regulated during *F. fujikuroi* infection.

## Discussion

Bakanae disease is emerging as a serious problem in rice not only in India but also in several countries across the world. In India alone, more than 20% incidence of disease has been reported from different states of the country (Bashyal et al., 2016a). To understand the dynamic nature of the genome of the fungal pathogen, whole genome sequence analysis of highly virulent pathogen is of great significance. The comparative analysis of pathogenic field isolate of *F. fujikuroi* from different locations of the world will help in understanding the fungal virulence spectrum. The information on the genome sequence of bakanae pathogen *F. fujikuroi* isolates though already available from China, Taiwan, and Korea, precise information about the secretory proteins including cell wall degrading enzymes is limited. Thus, the importance of high-quality genome sequencing and gene annotation is a critical tool for the research community. In this study, we sequenced, assembled and analyzed the whole genome of *F. fujikuroi* isolate “F250”. The total assembly size was 42.47 Mb, which was within the range of *Gibberella fujikuroi* genomes (33.5–49 Mb).

Repetitive elements have played an important role in pathogen evolution. The majority of the repetitive content in *F. fujikuroi* was made up of retrotransposon sequence. Retrotransposons use the mechanism of “copy and paste” to propagate, allowing for many copies to be inserted throughout the genome. Thus, retrotransposons make up the majority of repetitive content in this species. These data are similar to those found in other fungi, including the rice endophyte *Harpophora oryzae* (Xu et al., 2014), the corn leaf blight disease-causing *Cochliobolus heterostrophus* (Santana et al., 2014), the human pathogens *Sporothrix schenckii* and *Sporothrix brasiliensis* (Teixeira et al., 2014), where the composition of repetitive elements is primarily made up of retrotransposons.

Characterization of the secretome is helpful to the understanding of the mechanisms of pathogen virulence and host infection. A subset of secreted proteins from pathogen is expected to determine the progress and success of the infection. A secretome size of 574 proteins is predicted for *F. graminearum*, representing 4.2% of the predicted total gene repertoire (Brown et al., 2012). A total of 1,336 secretory proteins were predicted in the genome of *F. fujikuroi* isolate IMI58289 (Wiemann et al., 2013). However, the study on the role of secretome including plant cell wall degrading-enzymes have been limited to the identification of no. of genes in *F. fujikuroi.* Results indicated that secretome of *F. fujikuroi* consists of diverse proteins that function in an organized manner to suppress different aspects of plant immunity for causing disease successfully. Furthermore, publicly available expression data indicate that many of these genes are upregulated during host–pathogen interaction. The genome seems to be enriched with glycoside hydrolases (GHs), and CEs to breach the host cell barrier during plant–pathogen interactions. Most cellulose degrading enzymes are categorized within GH classes and abundance of GH3 and GH5 which can catalyze the degradation of cellulose, hemicellulose, and pectin suggesting that these enzymes play important roles in *F. fujikuroi* genome. GHs were also reported maximum in *Fusarium graminearum* (Zhao et al., 2013), *Rhizoctonia solani* AG11A (Rioux et al., 2011) *Nectria haematococca* (Coleman et al., 2009) and *F*. *oxysporum* (Chang et al., 2016). Further, *F. fujikuroi* has one GH67 and one GH36 genes that encode (α-galactosidases) that are deficient in most plat pathogens. Abundance of pectin degrading enzyme families CE8 (4 genes), PL1 (10 genes), PL3 (5 genes), PL9 (2 genes), GH28 (8 genes), and GH78 (1 gene) may help *F. fujikuroi* in colonization of root tissues. Presence of CAZymes families like CE5 (12 genes), GH7 (2 genes), GH10 (1 gene), GH12 (3 genes), GH36 (1 gene), GH53 (1 gene), GH62 (1 gene), PL1 (10 genes), and PL3 (5 genes) suggested that *F. fujikuroi* have abundant enzymes related to cutinases and plant biomass degradation.

The whole genome annotation and comparative genomic analyses of the genome sequence of *F. fujikuroi* isolate from India provided insight into its genetic make-up and phylogenetic placement. Results indicated that the *F. fujikuroi* isolate F250 from India is more similar to Taiwan isolate IMI58289. Further, isolate KSUX-10626 from United States and B14 from South Korea were grouped together in a single cluster, whereas, KSU3368 from Thailand and Taiwan isolate FGSC8932 are more similar. The bakanae pathogen *F. fujikuroi* belongs to Asian clade of *F. fujikuroi* species complex which could be the one of the reason for the more diversity in the Asian isolates. Further, an additional PKS cluster has been identified in *F. fujikuroi* isolates KSUX10626 and B14 which is not present in other isolates (Chiara et al., 2015). Based on hierarchical clustering of average identity of aligned genome regions, Chiara et al. (2015) also observed similar results. Genomic differences among fungal isolates may be due to variation in environmental factors, host factors, mating types, and other micro variations such as SNPs and repetitive DNA elements.

The expression analysis of secretory proteins (including CAZYmes) indicated that all the predicted genes of *F. fujikuroi* were detected in the infected hosts. Expression of the cell wall degrading enzymes CBM (genes 5178, 11810, 12667, 13528), PL (genes 3064, 5526), CE (gene 12149), GH (gene 12698), and genes related to increased virulence (gene 9849) were maximum at 7 or 10 days of inoculation. This increase can be attributed to the upregulation of pectin degrading enzymes consistent with the abundance of pectins in cell walls. Such expression profile also suggested that particular enzyme may have specific roles at different point of infection. Further, higher activation of cell wall degrading enzyme genes in the susceptible genotype compared to the resistant one at early stage of inoculation could be due to more colonization of *F. fujikuroi* in the susceptible genotype “Rasi” and subsequent activation of the cell wall degrading enzymes. Some of the genes like, 12587 (GH-72 family) and 1530 (AA-7 family) have shown more expression in resistant genotype compared to the susceptible one. Further expression of these genes were maximum at 30 days of inoculation. As maximum transcriptome for the resistance are expressed after 3-week post germination in *F. fujikuroi*–rice interaction (Matic et al., 2016), genes related to reduced virulence might be activated at later stage of inoculation in resistant genotype compared to susceptible. Matic et al. (2016) reported that systemic and local behavior of *F. fujikuroi* is related to the rice genotype during *F. fujikuroi*–rice interactions which could be further responsible for the changes in the no. of genes expressed and their expression level. Results suggested that the gene expression profiles are distinct at the different infection times and it is likely that the expression of specific genes encoding degradation enzymes progressively damaged the rice plants during infection.

## Conclusion

We have sequenced and analyzed genomes of *F. fujikuroi* from Indian subcontinent where rice is grown in large areas. The sequence information, comparative analysis and secretome analysis from this study will be valuable resource for functional genomics studies in *F. fujikuroi* genome of the pathogen from India (NCBI accession no. MBPO00000000) accelerate study of host–pathogen interaction and development of strategies for resistance breeding against bakanae disease in rice.

## Availability of Data and Materials

This Whole Genome Shotgun project has been deposited at DDBJ/ENA/GenBank under the accession MBPO00000000. The version described in this paper is version MBPO01000000.

## Author Contributions

All authors have read and approved the final manuscript. BB, RA, AKS, and TS involved in conceptualization of the project, study design, bio-informatics analyses, data compilation, manuscript writing, critical inputs and finalization of the manuscript; BB, KR, SS, DK, and HD were involved wet lab experiments and bio-informatics analysis, SG and AUS was involved in critical inputs and finalization of the manuscript.

## Conflict of Interest Statement

The authors declare that the research was conducted in the absence of any commercial or financial relationships that could be construed as a potential conflict of interest.

## Footnotes

1 https://www.ncbi.nlm.nih.gov/blast/

2 https://www.idtdna.com/Primerquest/Home/Index

## References

[B1] Bashyal B. M. Aggarwal R. (2013). Molecular identification of *Fusarium* spp. associated with bakanae disease of rice in India. *Ind. J. Agric. Sci.* 83 72–77.

[B2] Bashyal B. M. Aggarwal R. Banerjee S. Gupta S. Sharma S. (2014). “Pathogenicity, ecology and genetic diversity of the *Fusarium* spp. associated with an emerging bakanae disease of rice (*Oryza sativa* L.) in India,” in *Microbial Diversity and Biotechnology in Food Security India* eds Kharwar R. N. Upadhyay R. Dubey N. Raghuwanshi R. (New Delhi: Springer) 307–314.

[B3] Bashyal B. M. Aggarwal R. Sharma S. Gupta S. Rawat K. Singh D. (2016a). Occurrence, identification and pathogenicity of *Fusarium* species associated with bakanae disease of basmati rice in India. *Eur. J. Plant Pathol.* 144 457–466. 10.1007/s10658-015-0783-8

[B4] Bashyal B. M. Aggarwal R. Sharma S. Gupta S. Singh U. B. (2016b). Single and combined effects of three *Fusarium* species associated with rice seeds on the severity of bakanae disease of rice. *J. Plant Pathol.* 98 405–412.

[B5] BLAST (2016). Basic Local Alignment Search Tool at National Center for Biotechnology Information (NCBI). Available at: https://blast.ncbi.nlm.nih.gov/Blast.cgi

[B6] Blast2GO (2016). *High-quality Functional Annotation Up and Running within no Time.* Available at: http://www.blast2go.com/b2ghome/ [accessed June 10, 2016].

[B7] Brown N. A. Antoniw J. Hammond-Kosack K. E. (2012). The predicted secretome of the plant pathogenic fungus *Fusarium graminearum*: a refined comparative analysis. *PLOS ONE* 7:e33731. 10.1371/journal.pone.0033731 22493673 PMC3320895

[B8] Cazy (2016). *Carbohydrate-Active enZYmes Database.* Available at: http://www.cazy.org/ [accessed June 21, 2016].

[B9] Chang H. X. Yendrek C. R. Caetano-Anolles G. Hartman G. L. (2016). Genomic characterization of plant cell wall degrading enzymes and *in silico* analysis of xylanses and polygalacturonases of *Fusarium virguliforme*. *BMC Microbiol.* 16:147. 10.1186/s12866-016-0761-0 27405320 PMC4941037

[B10] Chiara M. Fanelli F. Mule G. Logrieco A. F. Pesole G. Leslie J. F. (2015). Genome sequencing of multiple isolates highlights sub-telomeric genomic diversity within *Fusarium fujikuroi*. *Genome Biol. Evol.* 7 3062–3069. 10.1093/gbe/evv198 26475319 PMC5635591

[B11] Cingolani P. Platts A. Wang L. L. Coon M. Nguyen T. Wnag L. (2012). A program for annotating and predicting the effects of single nucleotide polymorphisms, SnpEff, SNPs in the genome of *Drosophila melanogaster* strain w1118; iso-2; iso-3. *Fly* 6 80–92. 10.4161/fly.19695 22728672 PMC3679285

[B12] Coleman J. J. Rounsley S. D. Rodriguez-Carres M. Kuo A. Wasmann C. C. Grimwood J. (2009). The genome of *Nectria haematococca*: contribution of supernumerary chromosomes to gene expansion. *PLOS Genet.* 5:e1000618. 10.1371/journal.pgen.1000618 19714214 PMC2725324

[B13] Fiyaz R. A. Krishnan S. G. Rajashekara H. Yadav A. K. Bashyal B. M. Bhowmick P. K. (2014). Development of high throughput screening protocol and identification of novel sources of resistance against bakanae disease in rice (*Oryza sativa* L.). *Ind. J. Genet.* 74 414–422.

[B14] Fiyaz R. A. Yadav A. K. Krishnan S. G. Ellur R. K. Bashyal B. M. Grover N. (2016). Mapping quantitative trait loci responsible for resistance to bakanae disease in rice. *Rice* 9:45. 10.1186/s12284-016-0117-2 27620639 PMC5019990

[B15] Gupta A. K. Solanki I. S. Bashyal B. M. Singh Y. Srivastava K. (2015). Bakanae of rice-an emerging disease in Asia. *J. Anim. Plant Sci.* 25 1499–1514.

[B16] Huggett J. Dheda K. Bustin S. Zumla A. (2005). Real-time RT-PCR normalisation; strategies and considerations. *Genes Immun.* 6 279–284. 10.1038/sj.gene.6364190 15815687

[B17] Jeong H. Lee S. Choi G. J. Lee T. Yun S. H. (2013). Draft genome sequence of *Fusarium fujikuroi* B14 the causal agent of the bakanae disease of rice. *Genome Announc.* 1:e00035-13. 10.1128/genomeA.00035-13 23472226 PMC3587928

[B18] King R. Urban M. Hammond M. C. U. Pak K. H. Hammond K. E. (2015). The completed genome sequence of the pathogenic ascomycete fungus *Fusarium graminearum*. *BMC Genomics* 16:544. 10.1186/s12864-015-1756-1 26198851 PMC4511438

[B19] Kini K. R. Let V. Mathur S. B. (2002). Genetic variation in *Fusarium moniliforme* isolated from seeds of different host species from Burkina Faso based on random amplified polymorphic DNA analysis. *J. Phytopathol.* 150 209–212. 10.1046/j.1439-0434.2002.00739.x

[B20] Kurtz S. Phillippy A. Delcher A. L. Smoot M. Shumway M. Antonescu C. (2004). Versatile and open software for comparing large genomes. *Genome Biol.* 5:R12. 10.1186/gb-2004-5-2-r12 14759262 PMC395750

[B21] Leslie J. F. Dickman M. B. (1991). Fate of DNA encoding hygromycin resistance after meiosis in transformed strains of *Gibberella fujikuroi* (*Fusarium moniliforme*). *Appl. Environ. Microbiol.* 57 1423–1429. 1854200 10.1128/aem.57.5.1423-1429.1991 PMC182965

[B22] Livak K. J. Schmittgen T. D. (2001). Analysis of relative gene expression data using real-time quantitative PCR and the 2^-ΔΔC_T_^ method. *Methods* 25 402–408. 10.1006/meth.2001.1262 11846609

[B23] Matic S. Bagnaresi P. Biselli C. Orru L. Carneiro G. A. Siciliano I. (2016). Comparative transcriptome profiling of resistant and susceptible rice genotypes in response to the seedborne pathogen *Fusarium fujikuroi*. *BMC Genomics* 17:608. 10.1186/s12864-016-2925-6 27515776 PMC4981969

[B24] MISA (2016). *MISA - MIcroSAtellite Identification Tool.* Available at: http://pgrc.ipk-gatersleben.de/misa/ [accessed July 6, 2016].

[B25] Murray M. G. Thompson W. F. (1980). Rapid isolation of high molecular weight plant DNA. *Nucleic Acids Res.* 8 4321–4326. 10.1093/nar/8.19.4321 7433111 PMC324241

[B26] Ou S. H. (ed.) (1985). “Bakanae disease and foot rot,” in *Rice Diseases Survey* (Kew: Commonwealth Mycological Institute) 262–272.

[B27] PHI (2017). *Base-host Pathogen Interaction Database.* Available at: http://www.phi-base.org/ [accessed February 17, 2017].

[B28] Phobius (2016). *A Combined Transmembrane Topology and Signal Peptide Predictor.* Available at: phobius.sbc.su.se/data.html [accessed June 17, 2016].

[B29] PredGPI (2017). *Prediction Server*. Available at: http://gpcr.biocomp.unibo.it/predgpi/ [accessed February 23, 2017].

[B30] Rioux R. Manmathan H. Singh P. de los Reyes B. Jia Y. L. Tavantzis S. (2011). Comparative analysis of putative pathogenesis-related gene expression in two *Rhizoctonia solani* pathosystems. *Curr. Genet.* 57 391–408. 10.1007/s00294-011-0353-3 21909999

[B31] Saitou N. Nei M. (1987). The neighbor-joining method: a new method for reconstructing phylogenetic trees. *Mol. Biol. Evol.* 4 406–425. 3447015 10.1093/oxfordjournals.molbev.a040454

[B32] Santana M. F. Silva J. C. Mizubuti E. S. Araújo E. F. Condon B. J. Turgeon B. G. (2014). Characterization and potential evolutionary impact of transposable elements in the genome of *Cochliobolus heterostrophus*. *BMC Genomics* 15:536. 10.1186/1471-2164-15-536 24973942 PMC4112212

[B33] Saremi H. (2005). *Fusarium, Biology, Ecology and Taxonomy* 1st Edn. Tehran: Jehad Daneshgahi Press 153.

[B34] Sharma R. Cao P. Jung K. H. Sharma M. K. Ronald P. C. (2013). Construction of a rice glycoside hydrolase phylogenomic database and identification of targets for biofuel research. *Front. Plant Sci.* 4:330. 10.3389/fpls.2013.00330 23986771 PMC3752443

[B35] SignalP (2016). *SignalP4 1 Server.* Available at: http://www.cbs.dtu.dk/services/SignalP [accessed June 17, 2016].

[B36] Steenkamp E. T. Wingfield B. D. Coutinho T. A. Zeller K. A. Wingfield M. J. Walter F. O. (2000). PCR-based identification of MAT-1 and MAT-2 in the *Gibberella fujikuroi* species complex. *Appl. Environ. Microbiol.* 66 4378–4382. 10.1128/AEM.66.10.4378-4382.2000 11010886 PMC92312

[B37] Subbaiyan G. K. Waters D. L. E. Katiyar S. K. Sadananda A. R. Satyadev V. Henry R. (2012). Genome-wide DNA polymorphisms in elite indica rice inbreds discovered by whole-genome sequencing. *Plant Biotechnol. J.* 10 623–634. 10.1111/j.1467-7652.2011.00676.x 22222031

[B38] Sun S. K. Snyder W. C. (1981). “The bakanae disease of the rice plant,” in *Fusarium: Diseases, Biology and Taxonomy* eds Nelson P. E. Toussoun T. A. Cook R. J. (University Park, PA: The Pennsylvania University Press) 104–113.

[B39] TargetP (2016). *TargetP 1.1 Server.* Available at: http://www.cbs.dtu.dk/services/TargetP/ [accessed June 17, 2016].

[B40] Teixeira M. M. de Almeida L. G. Kubitschek-Barreira P. Alves F. L. Kioshima E. S. Abadio A. K. (2014). Comparative genomics of the major fungal agents of human and animal sporotrichosis: *Sporothrix schenckii* and *Sporothrix brasiliensis*. *BMC Genomics* 15:943. 10.1186/1471-2164-15-943 25351875 PMC4226871

[B41] TMHMM (2016). *Server v. 2.0: Prediction of Transmembrane Helices in Proteins.* Available at: http://www.cbs.dtu.dk/services/TMHMM/ [accessed June 18, 2016].

[B42] Webster R. K. Gunnell P. S. (1992). *Compendium of Rice Diseases.* St. Paul, MN: The American Phytopathological Society Press.

[B43] Wiemann P. Sieber C. M. von Bargen K. W. Studt L. Niehaus E. M. Espino J. J. (2013). Deciphering the cryptic genome: genome-wide analyses of the rice pathogen *Fusarium fujikuroi* reveal complex regulation of secondary metabolism and novel metabolites. *PLOS Pathog.* 9:e1003475. 10.1371/journal.ppat.1003475 23825955 PMC3694855

[B44] Winnenburg R. Baldwin T. K. Urban M. Rawlings C. Köhler J. Hammond-Kosack K. E. (2006). PHI-base: a new database for pathogen host interactions. *Nucleic Acids Res.* 34 D459–D464. 10.1093/nar/gkj047 16381911 PMC1347410

[B45] WoLFPSORT (2017). *Protein Subcellular Localization Prediction.* Available at: https://wolfpsort.hgc.jp [accessed February 23, 2017].

[B46] Xu X. H. Su Z. Z. Wang C. Kubicek C. P. Feng X. X. Mao L. J. (2014). The rice endophyte *Harpophora oryzae* genome reveals evolution from a pathogen to a mutualistic endophyte. *Sci. Rep.* 4:5783. 10.1038/srep05783 25048173 PMC4105740

[B47] Zhao Z. Liu H. Wang C. Xu J. R. (2013). Comparative analysis of fungal genomes reveals different plant cell wall degrading capacity in fungi. *BMC Genomics* 14:274. 10.1186/1471-2164-14-274 23617724 PMC3652786

