# Supplementary material for: Whole Genome Sequencing of Fusarium fujikuroi Provides Insight into the Role of Secretory Proteins and Cell Wall Degrading Enzymes in Causing Bakanae Disease of Rice
Source: Front Plant Sci. 2017 Nov 27;8:2013. doi: 10.3389/fpls.2017.02013 (PMC5711826; doi:10.3389/fpls.2017.02013)
Supplement: Supplementary file 1 [file Table_1.PDF]

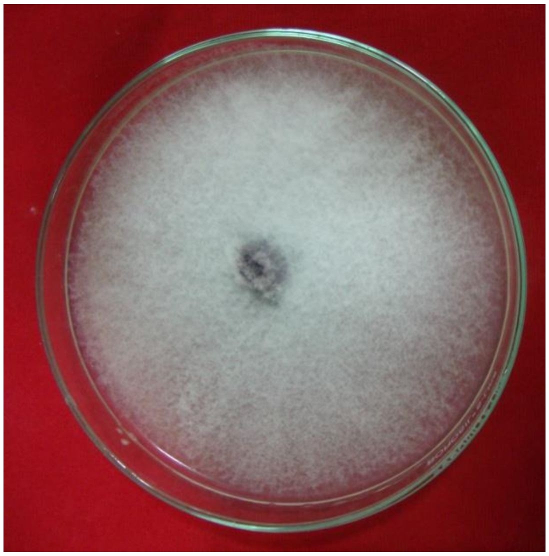

**Supplementary Fig. 1.** Culture plate of *F. fujikuroi* ( F250) isolate

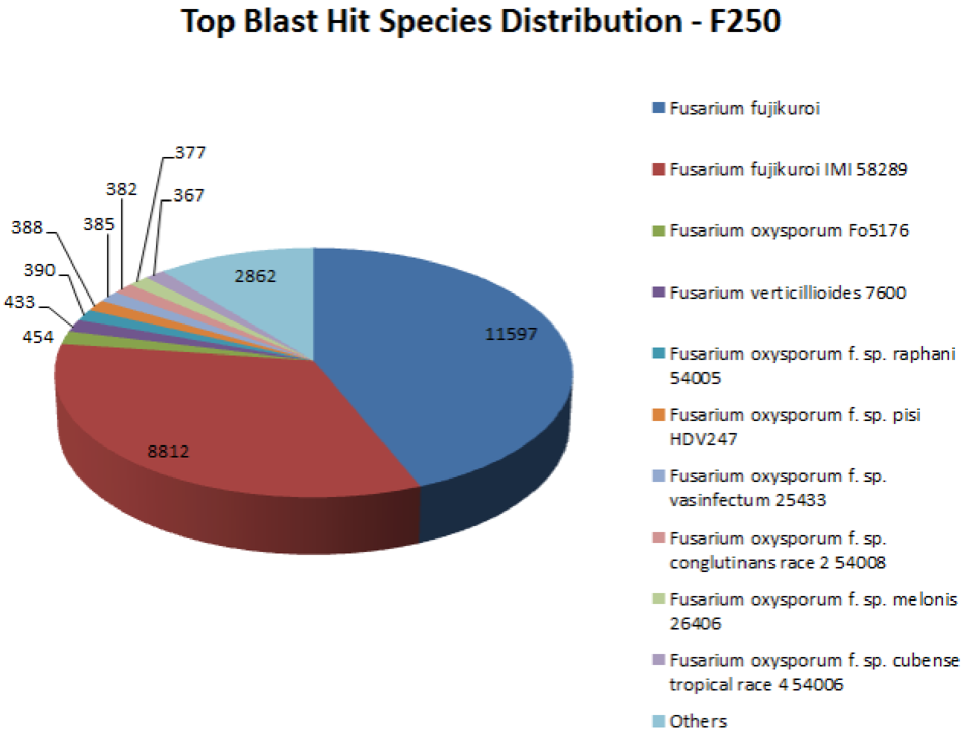


**Supplementary Fig. 2:** Top Blast hit species distribution of F250 isolate

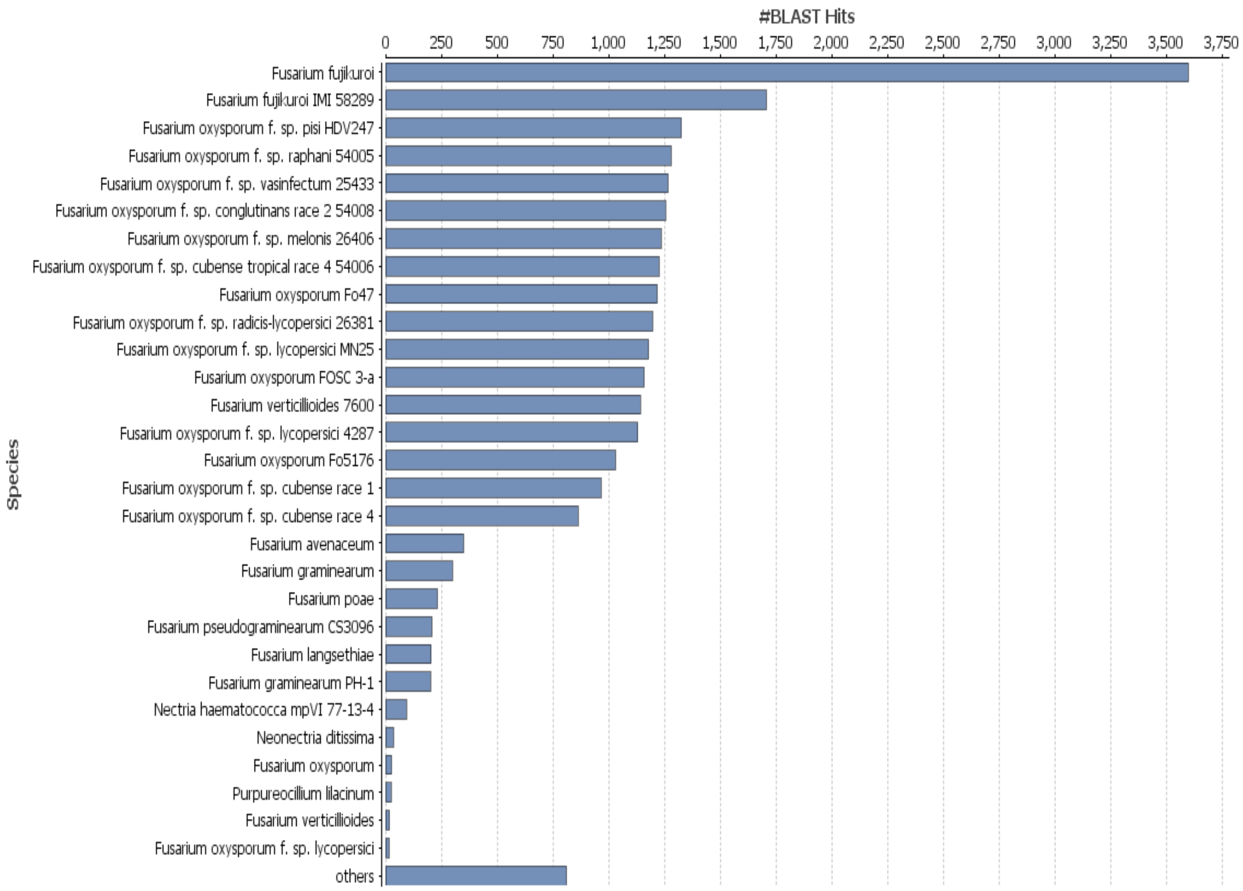


**Supplementary Fig. 3.** Blast hits of secretome of *F. fujikuroi* showing the closest species
**Table:** CAZyme analysis of secretome of *F. fujikuroi*

**Supplementary Table 1:** CAZyme analysis of secretome of *F. fujikuroi*

| Query | Evalue | Subject start | Subject end | Query start | Query end | Covered fraction | Subject |
|---|---|---|---|---|---|---|---|
| F250_g11052.t1 | 7.40E-56 | 442 | 742 | 38 | 357 | 0.318134 | AA1 |
| F250_g12074.t1 | 7.30E-86 | 441 | 737 | 123 | 431 | 0.313892 | AA1 |
| F250_g2953.t1 | 7.90E-15 | 14 | 114 | 114 | 215 | 0.392157 | AA2 |
| F250_g3266.t1 | 5.20E-47 | 2 | 254 | 186 | 434 | 0.988235 | AA2 |
| F250_g3634.t1 | 1.20E-26 | 6 | 181 | 46 | 222 | 0.686275 | AA2 |
| F250_g10844.t1 | 1.90E-82 | 85 | 400 | 26 | 602 | 0.509709 | AA3 |
| F250_g12209.t1 | 1.20E-72 | 85 | 401 | 23 | 637 | 0.511327 | AA3 |
| F250_g13464.t1 | 3.80E-85 | 86 | 400 | 41 | 622 | 0.508091 | AA3 |
| F250_g3024.t1 | 8.60E-73 | 87 | 397 | 21 | 634 | 0.501618 | AA3 |
| F250_g3415.t1 | 6.20E-78 | 83 | 401 | 32 | 665 | 0.514563 | AA3 |
| F250_g3436.t1 | 5.40E-89 | 85 | 403 | 16 | 589 | 0.514563 | AA3 |
| F250_g4657.t1 | 3.00E-85 | 84 | 403 | 31 | 579 | 0.516181 | AA3 |
| F250_g6827.t1 | 7.50E-76 | 79 | 400 | 31 | 602 | 0.519417 | AA3 |

| | | | | | | | |
|---|---|---|---|---|---|---|---|
| F250_g8350.t1 | 3.80E-95 | 84 | 398 | 28 | 607 | 0.508091 | AA3 |
| F250_g8679.t1 | 1.00E-79 | 89 | 401 | 23 | 638 | 0.504854 | AA3 |
| F250_g9069.t1 | 5.80E-40 | 88 | 397 | 19 | 535 | 0.5 | AA3 |
| F250_g1478.t1 | 9.80E-161 | 501 | 1026 | 211 | 1057 | 0.409836 | AA5 |
| F250_g3996.t1 | 1.50E-145 | 550 | 1025 | 104 | 678 | 0.370804 | AA5 |
| F250_g9564.t1 | 5.20E-147 | 511 | 1024 | 9 | 708 | 0.400468 | AA5 |
| F250_g9662.t1 | 9.80E-159 | 550 | 1025 | 103 | 680 | 0.370804 | AA5 |
| F250_g10432.t1 | 1.90E-45 | 13 | 198 | 116 | 320 | 0.40393 | AA7 |
| F250_g10851.t1 | 1.50E-49 | 19 | 218 | 121 | 328 | 0.434498 | AA7 |
| F250_g10962.t1 | 5.80E-51 | 19 | 218 | 77 | 275 | 0.434498 | AA7 |
| F250_g12034.t1 | 2.90E-53 | 12 | 217 | 61 | 266 | 0.447598 | AA7 |
| F250_g13412.t1 | 1.60E-92 | 5 | 458 | 54 | 491 | 0.989083 | AA7 |
| F250_g13527.t1 | 9.10E-53 | 4 | 454 | 69 | 505 | 0.982533 | AA7 |
| F250_g13530.t1 | 2.40E-53 | 5 | 456 | 80 | 511 | 0.984716 | AA7 |
| F250_g13535.t1 | 2.40E-45 | 8 | 213 | 175 | 393 | 0.447598 | AA7 |
| F250_g1530.t1 | 3.10E-50 | 12 | 211 | 59 | 258 | 0.434498 | AA7 |
| F250_g218.t1 | 8.20E-45 | 21 | 193 | 124 | 301 | 0.375546 | AA7 |
| F250_g2370.t1 | 1.70E-70 | 4 | 445 | 52 | 480 | 0.962882 | AA7 |
| F250_g3199.t1 | 4.50E-44 | 11 | 445 | 108 | 556 | 0.947598 | AA7 |
| F250_g5023.t1 | 7.10E-11 | 18 | 181 | 38 | 203 | 0.355895 | AA7 |
| F250_g5028.t1 | 1.60E-40 | 5 | 184 | 670 | 867 | 0.39083 | AA7 |
| F250_g5857.t1 | 1.70E-55 | 8 | 455 | 67 | 526 | 0.975983 | AA7 |
| F250_g5886.t1 | 6.20E-52 | 13 | 189 | 114 | 302 | 0.384279 | AA7 |
| F250_g8364.t1 | 3.50E-44 | 15 | 212 | 191 | 404 | 0.430131 | AA7 |
| F250_g8367.t1 | 2.60E-44 | 3 | 454 | 62 | 497 | 0.984716 | AA7 |
| F250_g8839.t1 | 1.40E-44 | 5 | 213 | 52 | 263 | 0.454148 | AA7 |
| F250_g12855.t1 | 4.20E-190 | 236 | 779 | 17 | 542 | 0.666258 | AA8 |
| F250_g2261.t1 | 0 | 7 | 815 | 6 | 832 | 0.991411 | AA8 |
| F250_g3216.t1 | 4.50E-264 | 16 | 781 | 11 | 757 | 0.93865 | AA8 |
| F250_g6996.t1 | 7.30E-196 | 234 | 775 | 18 | 545 | 0.663804 | AA8 |
| F250_g8356.t1 | 1.40E-265 | 8 | 794 | 4 | 764 | 0.964417 | AA8 |
| F250_g11494.t1 | 8.90E-63 | 2 | 219 | 7 | 222 | 0.986364 | AA9 |
| F250_g11625.t1 | 5.90E-57 | 1 | 219 | 4 | 226 | 0.990909 | AA9 |
| F250_g12419.t1 | 2.30E-65 | 2 | 220 | 8 | 210 | 0.990909 | AA9 |
| F250_g13348.t1 | 8.70E-56 | 3 | 184 | 9 | 195 | 0.822727 | AA9 |
| F250_g2784.t1 | 2.00E-65 | 3 | 218 | 11 | 238 | 0.977273 | AA9 |
| F250_g284.t1 | 1.30E-63 | 2 | 219 | 10 | 234 | 0.986364 | AA9 |
| F250_g3294.t1 | 9.00E-76 | 3 | 219 | 6 | 233 | 0.981818 | AA9 |
| F250_g4153.t1 | 9.00E-67 | 3 | 219 | 9 | 227 | 0.981818 | AA9 |
| F250_g433.t1 | 2.20E-70 | 2 | 219 | 10 | 234 | 0.986364 | AA9 |
| F250_g4367.t1 | 1.50E-53 | 2 | 219 | 6 | 231 | 0.986364 | AA9 |
| F250_g5881.t1 | 5.00E-65 | 2 | 219 | 9 | 225 | 0.986364 | AA9 |
| F250_g6401.t1 | 2.80E-64 | 3 | 219 | 11 | 233 | 0.981818 | AA9 |
| F250_g9483.t1 | 2.50E-56 | 2 | 219 | 8 | 228 | 0.986364 | AA9 |
| F250_g10200.t1 | 2.90E-15 | 1 | 29 | 374 | 402 | 0.965517 | CBM1 |
| F250_g11650.t1 | 1.20E-14 | 2 | 29 | 567 | 594 | 0.931034 | CBM1 |
| F250_g11810.t1 | 1.50E-16 | 2 | 29 | 22 | 49 | 0.931034 | CBM1 |
| F250_g1825.t1 | 4.80E-16 | 1 | 29 | 482 | 510 | 0.965517 | CBM1 |
| F250_g2579.t1 | 5.00E-13 | 1 | 29 | 517 | 545 | 0.965517 | CBM1 |
| F250_g4310.t1 | 1.10E-14 | 2 | 29 | 21 | 48 | 0.931034 | CBM1 |
| F250_g4371.t1 | 6.70E-13 | 2 | 29 | 30 | 57 | 0.931034 | CBM1 |
| F250_g9031.t1 | 5.00E-10 | 2 | 29 | 345 | 375 | 0.931034 | CBM1 |
| F250_g9760.t1 | 2.50E-11 | 2 | 29 | 365 | 393 | 0.931034 | CBM1 |

| | | | | | | | |
|---|---|---|---|---|---|---|---|
| F250_g12618.t1 | 6.20E-13 | 2 | 74 | 117 | 198 | 0.62069 | CBM16 |
| F250_g13285.t1 | 1.30E-07 | 1 | 102 | 90 | 206 | 0.87069 | CBM16 |
| F250_g2461.t1 | 1.50E-07 | 1 | 71 | 161 | 232 | 0.603448 | CBM16 |
| F250_g1031.t1 | 0.00017 | 8 | 35 | 24 | 51 | 0.710526 | CBM18 |
| F250_g12667.t1 | 2.80E-10 | 6 | 38 | 453 | 501 | 0.842105 | CBM18 |
| F250_g1723.t1 | 1.40E-08 | 1 | 36 | 26 | 61 | 0.921053 | CBM18 |
| F250_g3187.t1 | 1.40E-05 | 2 | 35 | 36 | 69 | 0.868421 | CBM18 |
| F250_g5178.t1 | 2.20E-07 | 2 | 35 | 52 | 84 | 0.868421 | CBM18 |
| F250_g640.t1 | 1.20E-06 | 7 | 35 | 47 | 76 | 0.736842 | CBM18 |
| F250_g3898.t1 | 0.00017 | 4 | 43 | 364 | 404 | 0.866667 | CBM19 |
| F250_g2530.t1 | 1.50E-24 | 2 | 88 | 484 | 572 | 0.955556 | CBM20 |
| F250_g5956.t1 | 8.80E-28 | 1 | 88 | 540 | 630 | 0.966667 | CBM20 |
| F250_g12428.t1 | 7.30E-08 | 3 | 75 | 145 | 219 | 0.549618 | CBM22 |
| F250_g12234.t1 | 0.00012 | 31 | 122 | 363 | 475 | 0.739837 | CBM35 |
| F250_g13145.t1 | 4.50E-08 | 6 | 122 | 425 | 553 | 0.943089 | CBM35 |
| F250_g9465.t1 | 1.10E-17 | 3 | 120 | 332 | 447 | 0.95122 | CBM35 |
| F250_g715.t1 | 8.20E-22 | 36 | 129 | 327 | 432 | 0.72093 | CBM38 |
| F250_g13523.t1 | 0.00026 | 1 | 65 | 400 | 470 | 0.507937 | CBM4 |
| F250_g13233.t1 | 1.60E-55 | 2 | 136 | 354 | 494 | 0.985294 | CBM42 |
| F250_g13128.t1 | 0.00075 | 1 | 38 | 1317 | 1362 | 0.925 | CBM50 |
| F250_g7549.t1 | 1.80E-06 | 1 | 28 | 45 | 73 | 0.675 | CBM50 |
| F250_g915.t1 | 7.30E-08 | 1 | 39 | 27 | 73 | 0.95 | CBM50 |
| F250_g4566.t1 | 0.00075 | 50 | 132 | 227 | 322 | 0.557823 | CBM57 |
| F250_g7027.t1 | 4.20E-40 | 16 | 137 | 325 | 449 | 0.876812 | CBM6 |
| F250_g11387.t1 | 0.00028 | 2 | 70 | 41 | 114 | 0.48227 | CBM61 |
| F250_g12830.t1 | 6.50E-07 | 2 | 74 | 103 | 175 | 0.510638 | CBM61 |
| F250_g12949.t1 | 1.50E-06 | 3 | 77 | 90 | 168 | 0.524823 | CBM61 |
| F250_g4544.t1 | 7.60E-22 | 3 | 71 | 243 | 313 | 0.871795 | CBM63 |
| F250_g561.t1 | 1.50E-10 | 3 | 69 | 149 | 217 | 0.846154 | CBM63 |
| F250_g5767.t1 | 9.40E-22 | 6 | 70 | 140 | 206 | 0.820513 | CBM63 |
| F250_g7149.t1 | 3.60E-05 | 13 | 154 | 194 | 329 | 0.909677 | CBM66 |
| F250_g10835.t1 | 7.20E-08 | 15 | 194 | 171 | 398 | 0.788546 | CE1 |
| F250_g10907.t1 | 1.90E-05 | 85 | 174 | 227 | 331 | 0.39207 | CE1 |
| F250_g12340.t1 | 7.10E-06 | 91 | 209 | 601 | 735 | 0.519824 | CE1 |
| F250_g12506.t1 | 9.20E-07 | 12 | 207 | 117 | 405 | 0.859031 | CE1 |
| F250_g12824.t1 | 5.50E-11 | 24 | 143 | 64 | 239 | 0.524229 | CE1 |
| F250_g13017.t1 | 0.00048 | 21 | 125 | 128 | 248 | 0.45815 | CE1 |
| F250_g13042.t1 | 2.60E-23 | 8 | 182 | 42 | 233 | 0.76652 | CE1 |
| F250_g1933.t1 | 5.40E-20 | 3 | 143 | 44 | 210 | 0.61674 | CE1 |
| F250_g2802.t1 | 9.20E-26 | 8 | 155 | 39 | 201 | 0.647577 | CE1 |
| F250_g4258.t1 | 2.50E-05 | 92 | 186 | 171 | 299 | 0.414097 | CE1 |
| F250_g5514.t1 | 1.10E-06 | 20 | 192 | 180 | 399 | 0.757709 | CE1 |
| F250_g8674.t1 | 7.70E-07 | 12 | 207 | 119 | 405 | 0.859031 | CE1 |
| F250_g9462.t1 | 1.80E-10 | 14 | 197 | 183 | 414 | 0.806167 | CE1 |
| F250_g9556.t1 | 2.80E-05 | 12 | 140 | 102 | 234 | 0.563877 | CE1 |
| F250_g985.t1 | 9.10E-05 | 24 | 143 | 98 | 259 | 0.524229 | CE1 |
| F250_g9863.t1 | 4.60E-07 | 28 | 178 | 83 | 268 | 0.660793 | CE1 |
| F250_g11474.t1 | 1.60E-34 | 75 | 236 | 90 | 268 | 0.472141 | CE10 |
| F250_g11646.t1 | 1.00E-26 | 64 | 179 | 81 | 238 | 0.337243 | CE10 |
| F250_g11808.t1 | 1.00E-34 | 82 | 333 | 109 | 370 | 0.73607 | CE10 |
| F250_g2121.t1 | 8.80E-07 | 88 | 209 | 143 | 296 | 0.354839 | CE10 |
| F250_g2834.t1 | 1.20E-30 | 76 | 203 | 98 | 246 | 0.372434 | CE10 |
| F250_g3764.t1 | 3.40E-29 | 62 | 333 | 46 | 298 | 0.794721 | CE10 |

| | | | | | | | |
|---|---|---|---|---|---|---|---|
| F250_g3851.t1 | 3.50E-31 | 70 | 187 | 90 | 223 | 0.343109 | CE10 |
| F250_g3980.t1 | 4.90E-31 | 69 | 186 | 113 | 250 | 0.343109 | CE10 |
| F250_g4019.t1 | 1.50E-31 | 75 | 188 | 94 | 218 | 0.331378 | CE10 |
| F250_g4155.t1 | 6.00E-25 | 76 | 209 | 150 | 310 | 0.390029 | CE10 |
| F250_g531.t1 | 2.20E-29 | 74 | 193 | 120 | 258 | 0.348974 | CE10 |
| F250_g549.t1 | 2.60E-19 | 28 | 298 | 13 | 279 | 0.791789 | CE10 |
| F250_g7840.t1 | 9.30E-35 | 75 | 182 | 109 | 230 | 0.313783 | CE10 |
| F250_g8029.t1 | 6.40E-26 | 75 | 185 | 106 | 228 | 0.322581 | CE10 |
| F250_g8227.t1 | 5.20E-29 | 75 | 207 | 104 | 244 | 0.387097 | CE10 |
| F250_g872.t1 | 2.10E-29 | 72 | 187 | 135 | 265 | 0.337243 | CE10 |
| F250_g9199.t1 | 6.10E-24 | 69 | 180 | 99 | 221 | 0.325513 | CE10 |
| F250_g9498.t1 | 1.40E-32 | 71 | 209 | 118 | 271 | 0.404692 | CE10 |
| F250_g3215.t1 | 1.10E-47 | 1 | 210 | 22 | 235 | 0.995238 | CE12 |
| F250_g8468.t1 | 9.90E-40 | 2 | 209 | 30 | 215 | 0.985714 | CE12 |
| F250_g9581.t1 | 1.10E-43 | 3 | 209 | 26 | 210 | 0.980952 | CE12 |
| F250_g12301.t1 | 5.80E-10 | 4 | 124 | 38 | 164 | 0.967742 | CE14 |
| F250_g10194.t1 | 8.30E-54 | 1 | 266 | 67 | 352 | 0.992509 | CE16 |
| F250_g11833.t1 | 1.00E-63 | 1 | 266 | 33 | 282 | 0.992509 | CE16 |
| F250_g1574.t1 | 4.10E-96 | 1 | 266 | 29 | 287 | 0.992509 | CE16 |
| F250_g3609.t1 | 1.60E-53 | 2 | 266 | 39 | 308 | 0.988764 | CE16 |
| F250_g4380.t1 | 1.20E-64 | 1 | 266 | 33 | 279 | 0.992509 | CE16 |
| F250_g6276.t1 | 4.50E-22 | 1 | 265 | 448 | 708 | 0.988764 | CE16 |
| F250_g222.t1 | 3.10E-49 | 1 | 207 | 128 | 339 | 0.985646 | CE2 |
| F250_g11048.t1 | 1.60E-46 | 1 | 194 | 483 | 681 | 0.994845 | CE3 |
| F250_g159.t1 | 2.20E-30 | 1 | 165 | 135 | 352 | 0.845361 | CE3 |
| F250_g2085.t1 | 5.00E-21 | 1 | 110 | 57 | 171 | 0.561856 | CE3 |
| F250_g2859.t1 | 8.50E-42 | 1 | 194 | 70 | 270 | 0.994845 | CE3 |
| F250_g6887.t1 | 1.60E-59 | 1 | 194 | 79 | 268 | 0.994845 | CE3 |
| F250_g10809.t1 | 6.60E-28 | 6 | 126 | 38 | 162 | 0.923077 | CE4 |
| F250_g11384.t1 | 1.00E-23 | 6 | 125 | 47 | 170 | 0.915385 | CE4 |
| F250_g11385.t1 | 1.00E-25 | 7 | 125 | 44 | 167 | 0.907692 | CE4 |
| F250_g3597.t1 | 1.30E-15 | 23 | 126 | 351 | 458 | 0.792308 | CE4 |
| F250_g4646.t1 | 2.70E-34 | 8 | 127 | 32 | 155 | 0.915385 | CE4 |
| F250_g11188.t1 | 7.40E-40 | 1 | 188 | 52 | 228 | 0.989418 | CE5 |
| F250_g12627.t1 | 0.00015 | 66 | 141 | 290 | 364 | 0.396825 | CE5 |
| F250_g1713.t1 | 3.20E-48 | 2 | 189 | 202 | 371 | 0.989418 | CE5 |
| F250_g3031.t1 | 7.40E-42 | 1 | 188 | 47 | 223 | 0.989418 | CE5 |
| F250_g3110.t1 | 0.00038 | 64 | 180 | 148 | 298 | 0.613757 | CE5 |
| F250_g3466.t1 | 4.10E-43 | 5 | 187 | 32 | 223 | 0.962963 | CE5 |
| F250_g3467.t1 | 2.40E-41 | 3 | 179 | 28 | 213 | 0.931217 | CE5 |
| F250_g4636.t1 | 4.00E-41 | 5 | 188 | 30 | 222 | 0.968254 | CE5 |
| F250_g470.t1 | 1.50E-42 | 1 | 189 | 22 | 227 | 0.994709 | CE5 |
| F250_g5739.t1 | 7.30E-22 | 1 | 119 | 68 | 180 | 0.624339 | CE5 |
| F250_g8418.t1 | 3.30E-43 | 1 | 187 | 30 | 232 | 0.984127 | CE5 |
| F250_g9023.t1 | 3.40E-35 | 2 | 188 | 24 | 228 | 0.984127 | CE5 |
| F250_g12718.t1 | 3.30E-55 | 4 | 280 | 51 | 381 | 0.958333 | CE8 |
| F250_g2742.t1 | 2.60E-45 | 4 | 166 | 47 | 212 | 0.5625 | CE8 |
| F250_g3639.t1 | 9.00E-26 | 2 | 269 | 372 | 637 | 0.927083 | CE8 |
| F250_g5510.t1 | 4.00E-78 | 3 | 275 | 27 | 307 | 0.944444 | CE8 |
| F250_g61.t1 | 3.10E-102 | 4 | 428 | 161 | 619 | 0.988345 | GH1 |
| F250_g9482.t1 | 6.20E-102 | 2 | 303 | 29 | 325 | 0.993399 | GH10 |
| F250_g10812.t1 | 9.50E-91 | 5 | 331 | 55 | 405 | 0.981928 | GH105 |
| F250_g6926.t1 | 8.10E-101 | 6 | 332 | 32 | 378 | 0.981928 | GH105 |

| | | | | | | | |
|---|---|---|---|---|---|---|---|
| F250_g7025.t1 | 1.20E-94 | 11 | 331 | 43 | 383 | 0.963855 | GH105 |
| F250_g12381.t1 | 1.40E-69 | 5 | 515 | 32 | 645 | 0.618932 | GH106 |
| F250_g12263.t1 | 2.60E-08 | 61 | 114 | 55 | 108 | 0.420635 | GH109 |
| F250_g6295.t1 | 3.40E-11 | 2 | 122 | 9 | 130 | 0.952381 | GH109 |
| F250_g11560.t1 | 1.70E-70 | 1 | 176 | 51 | 226 | 0.988701 | GH11 |
| F250_g314.t1 | 3.50E-75 | 1 | 176 | 44 | 219 | 0.988701 | GH11 |
| F250_g4375.t1 | 3.30E-75 | 2 | 176 | 54 | 228 | 0.983051 | GH11 |
| F250_g9172.t1 | 3.20E-50 | 1 | 190 | 59 | 277 | 0.994737 | GH114 |
| F250_g2170.t1 | 2.30E-236 | 12 | 596 | 44 | 699 | 0.837877 | GH115 |
| F250_g10978.t1 | 2.80E-24 | 2 | 141 | 189 | 338 | 0.891026 | GH12 |
| F250_g3184.t1 | 1.60E-37 | 2 | 143 | 101 | 236 | 0.903846 | GH12 |
| F250_g415.t1 | 1.10E-25 | 20 | 156 | 181 | 324 | 0.871795 | GH12 |
| F250_g10972.t1 | 1.10E-129 | 1 | 402 | 86 | 496 | 0.997512 | GH125 |
| F250_g6707.t1 | 1.30E-156 | 1 | 402 | 70 | 497 | 0.997512 | GH125 |
| F250_g9632.t1 | 1.60E-162 | 1 | 402 | 87 | 523 | 0.997512 | GH125 |
| F250_g2312.t1 | 3.20E-39 | 163 | 522 | 164 | 551 | 0.685115 | GH127 |
| F250_g10018.t1 | 6.90E-55 | 7 | 224 | 175 | 387 | 0.96875 | GH128 |
| F250_g10121.t1 | 1.50E-57 | 4 | 187 | 49 | 237 | 0.816964 | GH128 |
| F250_g5074.t1 | 1.30E-63 | 4 | 224 | 42 | 280 | 0.982143 | GH128 |
| F250_g13228.t1 | 5.60E-64 | 13 | 297 | 59 | 345 | 0.949833 | GH13 |
| F250_g8182.t1 | 3.70E-67 | 1 | 249 | 35 | 290 | 0.972549 | GH131 |
| F250_g7892.t1 | 6.00E-83 | 43 | 301 | 45 | 307 | 0.851485 | GH132 |
| F250_g9174.t1 | 1.00E-98 | 8 | 303 | 132 | 434 | 0.973597 | GH132 |
| F250_g10350.t1 | 1.20E-22 | 43 | 189 | 83 | 319 | 0.772487 | GH16 |
| F250_g11056.t1 | 1.30E-38 | 3 | 189 | 33 | 284 | 0.984127 | GH16 |
| F250_g11534.t1 | 9.90E-36 | 2 | 189 | 30 | 284 | 0.989418 | GH16 |
| F250_g11864.t1 | 1.90E-22 | 59 | 186 | 80 | 240 | 0.671958 | GH16 |
| F250_g12026.t1 | 1.60E-20 | 12 | 186 | 97 | 306 | 0.920635 | GH16 |
| F250_g13219.t1 | 1.10E-23 | 10 | 189 | 49 | 259 | 0.94709 | GH16 |
| F250_g5820.t1 | 8.60E-37 | 4 | 189 | 34 | 283 | 0.978836 | GH16 |
| F250_g6166.t1 | 1.50E-24 | 6 | 176 | 48 | 224 | 0.899471 | GH16 |
| F250_g7064.t1 | 4.60E-34 | 8 | 189 | 44 | 299 | 0.957672 | GH16 |
| F250_g7116.t1 | 6.60E-23 | 52 | 176 | 78 | 219 | 0.656085 | GH16 |
| F250_g791.t1 | 5.60E-20 | 55 | 176 | 84 | 222 | 0.640212 | GH16 |
| F250_g8132.t1 | 6.50E-22 | 3 | 188 | 78 | 291 | 0.978836 | GH16 |
| F250_g8335.t1 | 1.20E-16 | 23 | 176 | 82 | 314 | 0.809524 | GH16 |
| F250_g9032.t1 | 1.40E-40 | 2 | 189 | 30 | 277 | 0.989418 | GH16 |
| F250_g9265.t1 | 1.70E-14 | 89 | 189 | 86 | 225 | 0.529101 | GH16 |
| F250_g10637.t1 | 5.30E-25 | 8 | 298 | 89 | 333 | 0.932476 | GH17 |
| F250_g3061.t1 | 3.20E-17 | 26 | 285 | 62 | 291 | 0.832797 | GH17 |
| F250_g4186.t1 | 1.90E-16 | 73 | 310 | 105 | 310 | 0.762058 | GH17 |
| F250_g8930.t1 | 2.50E-06 | 16 | 111 | 368 | 464 | 0.305466 | GH17 |
| F250_g9583.t1 | 2.00E-31 | 19 | 311 | 50 | 302 | 0.938907 | GH17 |
| F250_g1577.t1 | 6.70E-52 | 4 | 290 | 21 | 422 | 0.966216 | GH18 |
| F250_g2026.t1 | 4.90E-74 | 3 | 286 | 323 | 672 | 0.956081 | GH18 |
| F250_g8046.t1 | 2.40E-59 | 27 | 283 | 49 | 404 | 0.864865 | GH18 |
| F250_g10810.t1 | 1.90E-56 | 35 | 502 | 32 | 708 | 0.621011 | GH2 |
| F250_g8389.t1 | 2.40E-100 | 23 | 412 | 53 | 500 | 0.517287 | GH2 |
| F250_g8900.t1 | 2.30E-87 | 7 | 336 | 213 | 558 | 0.976261 | GH20 |
| F250_g9403.t1 | 9.20E-61 | 6 | 336 | 169 | 509 | 0.979228 | GH20 |
| F250_g2113.t1 | 1.20E-36 | 3 | 137 | 101 | 241 | 0.978102 | GH24 |
| F250_g1109.t1 | 1.30E-22 | 254 | 374 | 234 | 351 | 0.32 | GH27 |
| F250_g6817.t1 | 1.40E-63 | 121 | 370 | 109 | 338 | 0.664 | GH27 |

| | | | | | | | |
|---|---|---|---|---|---|---|---|
| F250_g12000.t1 | 7.10E-35 | 69 | 306 | 191 | 450 | 0.729231 | GH28 |
| F250_g12698.t1 | 5.30E-65 | 9 | 321 | 74 | 425 | 0.96 | GH28 |
| F250_g2743.t1 | 2.20E-62 | 8 | 323 | 55 | 393 | 0.969231 | GH28 |
| F250_g2790.t1 | 4.30E-70 | 5 | 317 | 40 | 365 | 0.96 | GH28 |
| F250_g543.t1 | 8.60E-48 | 70 | 309 | 149 | 396 | 0.735385 | GH28 |
| F250_g8021.t1 | 4.80E-71 | 4 | 315 | 54 | 376 | 0.956923 | GH28 |
| F250_g8425.t1 | 3.20E-47 | 7 | 321 | 66 | 375 | 0.966154 | GH28 |
| F250_g9840.t1 | 5.20E-73 | 12 | 323 | 83 | 437 | 0.956923 | GH28 |
| F250_g12492.t1 | 2.30E-82 | 26 | 346 | 149 | 512 | 0.924855 | GH29 |
| F250_g7523.t1 | 2.10E-86 | 19 | 345 | 21 | 397 | 0.942197 | GH29 |
| F250_g12088.t1 | 3.40E-63 | 4 | 216 | 94 | 309 | 0.981481 | GH3 |
| F250_g12904.t1 | 1.70E-57 | 3 | 216 | 100 | 321 | 0.986111 | GH3 |
| F250_g137.t1 | 2.90E-61 | 3 | 215 | 75 | 324 | 0.981481 | GH3 |
| F250_g2117.t1 | 9.40E-55 | 4 | 187 | 88 | 278 | 0.847222 | GH3 |
| F250_g3997.t1 | 2.40E-42 | 5 | 216 | 76 | 263 | 0.976852 | GH3 |
| F250_g5575.t1 | 1.40E-62 | 8 | 215 | 115 | 333 | 0.958333 | GH3 |
| F250_g5611.t1 | 1.70E-58 | 3 | 216 | 98 | 321 | 0.986111 | GH3 |
| F250_g7570.t1 | 6.80E-56 | 12 | 213 | 138 | 368 | 0.930556 | GH3 |
| F250_g8408.t1 | 3.50E-49 | 7 | 215 | 113 | 334 | 0.962963 | GH3 |
| F250_g10180.t1 | 2.20E-85 | 7 | 415 | 27 | 482 | 0.978417 | GH30 |
| F250_g13135.t1 | 1.50E-66 | 14 | 415 | 20 | 430 | 0.961631 | GH30 |
| F250_g361.t1 | 1.90E-146 | 2 | 427 | 366 | 814 | 0.995316 | GH31 |
| F250_g12221.t1 | 1.20E-86 | 1 | 289 | 45 | 354 | 0.982935 | GH32 |
| F250_g716.t1 | 1.20E-73 | 1 | 287 | 41 | 355 | 0.976109 | GH32 |
| F250_g12827.t1 | 3.80E-93 | 1 | 303 | 48 | 378 | 0.983713 | GH35 |
| F250_g8399.t1 | 6.40E-89 | 2 | 304 | 45 | 390 | 0.983713 | GH35 |
| F250_g2447.t1 | 1.30E-239 | 2 | 687 | 37 | 727 | 0.99564 | GH36 |
| F250_g4686.t1 | 3.30E-158 | 2 | 490 | 51 | 626 | 0.99389 | GH37 |
| F250_g11324.t1 | 5.20E-33 | 11 | 218 | 28 | 418 | 0.834677 | GH43 |
| F250_g1138.t1 | 1.90E-35 | 1 | 244 | 21 | 316 | 0.979839 | GH43 |
| F250_g11469.t1 | 4.20E-15 | 13 | 211 | 79 | 325 | 0.798387 | GH43 |
| F250_g12828.t1 | 2.20E-19 | 10 | 209 | 153 | 403 | 0.802419 | GH43 |
| F250_g12899.t1 | 7.50E-44 | 1 | 247 | 29 | 301 | 0.991935 | GH43 |
| F250_g13136.t1 | 2.10E-23 | 8 | 208 | 88 | 341 | 0.806452 | GH43 |
| F250_g13425.t1 | 1.70E-55 | 4 | 244 | 31 | 314 | 0.967742 | GH43 |
| F250_g13426.t1 | 7.70E-54 | 5 | 247 | 28 | 312 | 0.975806 | GH43 |
| F250_g13536.t1 | 2.70E-40 | 3 | 228 | 39 | 308 | 0.907258 | GH43 |
| F250_g2748.t1 | 3.20E-57 | 1 | 248 | 27 | 304 | 0.995968 | GH43 |
| F250_g6991.t1 | 3.30E-22 | 2 | 247 | 23 | 293 | 0.987903 | GH43 |
| F250_g7028.t1 | 3.60E-45 | 1 | 247 | 19 | 286 | 0.991935 | GH43 |
| F250_g824.t1 | 3.80E-43 | 5 | 247 | 29 | 343 | 0.975806 | GH43 |
| F250_g9121.t1 | 1.90E-30 | 13 | 226 | 67 | 293 | 0.858871 | GH43 |
| F250_g10062.t1 | 3.60E-163 | 2 | 445 | 104 | 606 | 0.993274 | GH47 |
| F250_g12829.t1 | 3.70E-128 | 1 | 445 | 60 | 539 | 0.995516 | GH47 |
| F250_g1903.t1 | 2.70E-109 | 1 | 446 | 59 | 536 | 0.997758 | GH47 |
| F250_g5370.t1 | 8.00E-156 | 1 | 446 | 103 | 566 | 0.997758 | GH47 |
| F250_g78.t1 | 1.70E-133 | 2 | 409 | 95 | 518 | 0.912556 | GH47 |
| F250_g9945.t1 | 7.80E-161 | 2 | 446 | 117 | 605 | 0.995516 | GH47 |
| F250_g8570.t1 | 9.10E-183 | 5 | 548 | 24 | 598 | 0.989071 | GH49 |
| F250_g11832.t1 | 3.50E-38 | 10 | 252 | 20 | 336 | 0.88 | GH5 |
| F250_g12030.t1 | 4.50E-20 | 13 | 233 | 22 | 313 | 0.8 | GH5 |
| F250_g12127.t1 | 3.80E-15 | 93 | 240 | 119 | 315 | 0.534545 | GH5 |
| F250_g12625.t1 | 7.70E-23 | 30 | 253 | 132 | 421 | 0.810909 | GH5 |

| | | | | | | | |
|---|---|---|---|---|---|---|---|
| F250_g12656.t1 | 3.10E-35 | 6 | 235 | 34 | 335 | 0.832727 | GH5 |
| F250_g1731.t1 | 6.10E-28 | 28 | 244 | 81 | 366 | 0.785455 | GH5 |
| F250_g2431.t1 | 4.40E-28 | 34 | 244 | 52 | 298 | 0.763636 | GH5 |
| F250_g5648.t1 | 1.10E-32 | 24 | 251 | 102 | 409 | 0.825455 | GH5 |
| F250_g8032.t1 | 8.30E-35 | 19 | 236 | 82 | 373 | 0.789091 | GH5 |
| F250_g9063.t1 | 1.80E-18 | 9 | 240 | 30 | 336 | 0.84 | GH5 |
| F250_g96.t1 | 2.50E-40 | 14 | 253 | 55 | 336 | 0.869091 | GH5 |
| F250_g551.t1 | 3.30E-68 | 116 | 542 | 206 | 652 | 0.67619 | GH51 |
| F250_g13037.t1 | 4.50E-93 | 1 | 342 | 21 | 345 | 0.997076 | GH53 |
| F250_g7499.t1 | 8.20E-301 | 6 | 739 | 65 | 810 | 0.990541 | GH55 |
| F250_g7508.t1 | 2.70E-220 | 10 | 662 | 15 | 687 | 0.881081 | GH55 |
| F250_g8580.t1 | 5.50E-128 | 1 | 278 | 25 | 293 | 0.996403 | GH62 |
| F250_g6502.t1 | 4.30E-33 | 319 | 565 | 587 | 798 | 0.431579 | GH63 |
| F250_g1617.t1 | 8.70E-98 | 2 | 367 | 69 | 441 | 0.99455 | GH64 |
| F250_g4370.t1 | 1.80E-269 | 1 | 669 | 18 | 697 | 0.998505 | GH67 |
| F250_g4334.t1 | 1.70E-151 | 5 | 414 | 23 | 416 | 0.985542 | GH7 |
| F250_g5700.t1 | 7.40E-73 | 5 | 414 | 23 | 429 | 0.985542 | GH7 |
| F250_g12587.t1 | 3.70E-116 | 4 | 311 | 17 | 332 | 0.983974 | GH72 |
| F250_g7695.t1 | 3.50E-124 | 3 | 311 | 28 | 328 | 0.987179 | GH72 |
| F250_g5089.t1 | 4.00E-82 | 8 | 220 | 12 | 254 | 0.963636 | GH75 |
| F250_g5649.t1 | 1.70E-86 | 3 | 219 | 7 | 231 | 0.981818 | GH75 |
| F250_g10427.t1 | 6.00E-61 | 14 | 248 | 31 | 292 | 0.653631 | GH76 |
| F250_g13358.t1 | 1.20E-90 | 12 | 346 | 29 | 396 | 0.932961 | GH76 |
| F250_g1523.t1 | 1.90E-54 | 41 | 326 | 83 | 498 | 0.796089 | GH76 |
| F250_g1670.t1 | 5.90E-55 | 125 | 502 | 404 | 810 | 0.748016 | GH78 |
| F250_g12278.t1 | 2.00E-72 | 5 | 400 | 41 | 418 | 0.868132 | GH79 |
| F250_g10981.t1 | 1.10E-179 | 5 | 622 | 70 | 742 | 0.991961 | GH81 |
| F250_g3011.t1 | 1.60E-123 | 14 | 622 | 55 | 703 | 0.977492 | GH81 |
| F250_g7793.t1 | 2.70E-209 | 11 | 622 | 172 | 847 | 0.982315 | GH81 |
| F250_g13333.t1 | 2.50E-113 | 1 | 300 | 49 | 351 | 0.973941 | GH93 |
| F250_g4038.t1 | 1.30E-107 | 1 | 306 | 57 | 361 | 0.993485 | GH93 |
| F250_g5731.t1 | 2.40E-114 | 1 | 307 | 40 | 354 | 0.996743 | GH93 |
| F250_g6987.t1 | 1.00E-85 | 3 | 298 | 48 | 350 | 0.960912 | GH93 |
| F250_g8685.t1 | 8.80E-154 | 6 | 597 | 26 | 613 | 0.81856 | GH95 |
| F250_g3756.t1 | 8.50E-127 | 1 | 272 | 143 | 413 | 0.992674 | GT15 |
| F250_g4607.t1 | 2.00E-120 | 2 | 272 | 75 | 352 | 0.989011 | GT15 |
| F250_g4852.t1 | 4.30E-59 | 3 | 264 | 38 | 340 | 0.956044 | GT15 |
| F250_g13374.t1 | 1.00E-79 | 4 | 282 | 63 | 355 | 0.978873 | GT17 |
| F250_g4684.t1 | 1.90E-81 | 4 | 284 | 67 | 364 | 0.985915 | GT17 |
| F250_g11631.t1 | 1.70E-34 | 1 | 106 | 116 | 254 | 0.625 | GT2 |
| F250_g928.t1 | 5.20E-159 | 32 | 473 | 173 | 628 | 0.928421 | GT20 |
| F250_g10632.t1 | 1.40E-135 | 1 | 248 | 1167 | 1414 | 0.995968 | GT24 |
| F250_g5386.t1 | 4.90E-08 | 53 | 116 | 402 | 468 | 0.328125 | GT31 |
| F250_g8177.t1 | 5.30E-20 | 9 | 89 | 83 | 159 | 0.888889 | GT32 |
| F250_g9371.t1 | 5.30E-140 | 3 | 424 | 44 | 459 | 0.990588 | GT33 |
| F250_g2572.t1 | 6.20E-08 | 13 | 164 | 155 | 289 | 0.613821 | GT34 |
| F250_g2836.t1 | 3.60E-06 | 10 | 97 | 252 | 334 | 0.353659 | GT34 |
| F250_g3627.t1 | 3.40E-06 | 35 | 192 | 147 | 290 | 0.638211 | GT34 |
| F250_g4169.t1 | 4.40E-37 | 47 | 236 | 128 | 323 | 0.768293 | GT34 |
| F250_g5721.t1 | 1.40E-08 | 28 | 109 | 148 | 223 | 0.329268 | GT34 |
| F250_g4486.t1 | 6.60E-25 | 4 | 151 | 331 | 490 | 0.91875 | GT4 |
| F250_g322.t1 | 2.00E-12 | 38 | 199 | 69 | 219 | 0.555172 | GT54 |
| F250_g10713.t1 | 4.50E-104 | 5 | 268 | 77 | 341 | 0.981343 | GT62 |

| | | | | | | | |
|---|---|---|---|---|---|---|---|
| F250_g5769.t1 | 1.00E-68 | 2 | 239 | 173 | 411 | 0.991632 | GT69 |
| F250_g6973.t1 | 7.00E-69 | 2 | 238 | 141 | 379 | 0.987448 | GT69 |
| F250_g6096.t1 | 4.80E-49 | 3 | 264 | 156 | 431 | 0.988636 | GT71 |
| F250_g12347.t1 | 3.10E-25 | 26 | 254 | 43 | 278 | 0.88716 | GT8 |
| F250_g4063.t1 | 4.30E-21 | 30 | 254 | 57 | 289 | 0.871595 | GT8 |
| F250_g9388.t1 | 1.10E-24 | 22 | 254 | 130 | 375 | 0.902724 | GT8 |
| F250_g10979.t1 | 1.40E-52 | 21 | 201 | 110 | 297 | 0.891089 | PL1 |
| F250_g12626.t1 | 1.80E-50 | 20 | 201 | 108 | 296 | 0.89604 | PL1 |
| F250_g12831.t1 | 2.00E-59 | 10 | 201 | 62 | 260 | 0.945545 | PL1 |
| F250_g13061.t1 | 2.90E-52 | 20 | 200 | 111 | 297 | 0.891089 | PL1 |
| F250_g174.t1 | 5.00E-53 | 22 | 199 | 72 | 256 | 0.876238 | PL1 |
| F250_g2106.t1 | 2.10E-49 | 21 | 201 | 111 | 296 | 0.891089 | PL1 |
| F250_g3064.t1 | 8.30E-32 | 19 | 199 | 65 | 246 | 0.891089 | PL1 |
| F250_g3645.t1 | 1.20E-56 | 29 | 201 | 85 | 263 | 0.851485 | PL1 |
| F250_g539.t1 | 5.70E-50 | 18 | 201 | 105 | 294 | 0.905941 | PL1 |
| F250_g789.t1 | 1.40E-57 | 18 | 200 | 75 | 262 | 0.90099 | PL1 |
| F250_g10426.t1 | 7.40E-69 | 5 | 197 | 44 | 234 | 0.974619 | PL3 |
| F250_g12832.t1 | 8.80E-67 | 3 | 188 | 58 | 242 | 0.939086 | PL3 |
| F250_g1598.t1 | 9.80E-69 | 2 | 196 | 41 | 228 | 0.984772 | PL3 |
| F250_g3207.t1 | 9.50E-73 | 1 | 197 | 27 | 216 | 0.994924 | PL3 |
| F250_g5571.t1 | 2.70E-74 | 3 | 197 | 24 | 213 | 0.984772 | PL3 |
| F250_g1887.t1 | 3.50E-131 | 2 | 567 | 14 | 529 | 0.996473 | PL4 |
| F250_g6409.t1 | 4.60E-168 | 4 | 567 | 21 | 655 | 0.992945 | PL4 |
| F250_g2539.t1 | 2.30E-99 | 10 | 369 | 14 | 386 | 0.959893 | PL9 |
| F250_g5526.t1 | 4.20E-110 | 9 | 370 | 17 | 383 | 0.965241 | PL9 |

**Supplementary Table 2: Secretary proteins selected for the real time PCR showing maximum identity to NCBI database with accession no. and their description**

| Protein | Description/Family | Identity | Accession no. |
|---|---|---|---|
| F250_g1530.t1 | 6-hydroxy-d-nicotine oxidase [*Fusarium fujikuroi*]/AA-7 Family | 100% | KLO99162.1 |
| F250_g1593.t1 | related to pectinesterase [*Fusarium fujikuroi* IMI 58289]/ PL-3 Family | 99% | CCT74155.1 |
| F250_g3064.t1 | probable pectate lyase C [*Fusarium fujikuroi* IMI 58289]/ PL-1 Family | 100% | CCT64558.1 |
| F250_g4367.t1 | related to cellulose binding protein CEL1 [*Fusarium fujikuroi* IMI 58289] | 100% | CCT74544.1 |
| F250_g5178.t1 | probable chitin binding protein [*Fusarium fujikuroi* IMI 58289]/ CBM-18 Family | 94% | CCT73603.1 |
| F250_g5526.t1 | pectatelyase L precursor [*Fusarium fujikuroi*]/ PL-9 | 100% | KLP18119.1 |

| | | | |
|---|---|---|---|
| | Family | | |
| F250_g9483.t1 | Uncharacterized protein LW93_4415 [*Fusarium fujikuroi*]/ Glycosyl hydrolase family 61 | 100% | KLO79092.1 |
| F250_g9849.t1 | uncharacterized protein FFUJ_12628 [*Fusarium fujikuroi* IMI 58289]/ Necrosis inducing protein | 100% | CCT72736.1 |
| F250_g9762.t1 | Uncharacterized protein LW93_12242 [*Fusarium fujikuroi*] Cytochrome P450 | 99% | KLO88829.1 |
| F250_g11810.t1 | probable Putative endoglucanase type F [*Fusarium fujikuroi* IMI 58289]/ CBM-1 Family | 100% | CCT67762.1 |
| F250_g12149.t1 | cellulose binding protein CEL1 [*Fusarium fujikuroi* IMI 58289]/ CE-1 Family | 100% | KLP20144.1 |
| F250_g12587.t1 | probable beta (1-3) glucanosyltransferase [*Fusarium fujikuroi* IMI 58289]/ GH-72 Family | 100% | CCT67518.1 |
| F250_g12667.t1 | related to RF2 protein [*Fusarium fujikuroi* IMI 58289]/ CBM-18 Family | 100% | CCT65379.1 |
| F250_g12698.t1 | probable exo polygalacturonase [*Fusarium fujikuroi* IMI 58289]/GH-28 Family | 100% | CCT65422.1 |
| F250_g13285.t1 | uncharacterized protein FFUJ_06869 [*Fusarium fujikuroi* IMI 58289]/ CBM-16 Family | 90% | CCT68106.1 |
| F250_g13528.t1 | related to O-methylsterigmatocystin oxidoreductase [*Fusarium fujikuroi* IMI 58289] /CBM-16 Family | 99% | CCT74953.1 |

**Supplementary Table 3:** genes and primers used for the real time PCR based analysis

| **S. no** | **Gene** | **Forward** | **Reverse** | **length** |
|---|---|---|---|---|
| 1. | g1530 | AGCATACAAAGACTGGACCG | TCTTGAAGGTGATGGCGAAC | 269 |
| 2. | g1593 | AATCCTTGTGCCTCTTACGAC | CGAATGTTGCTGTACCCAATG | 247 |
| 3. | g3064 | ACTTCTACGACTCAAACCGC | CTGTGCAGTGAATTTCCAACG | 178 |

| | | | | |
|---|---|---|---|---|
| 4. | g4367 | ATGATCTTGAACCAGTCACCG | ACCAAGTACAACCCCATCAAG | 272 |
| 5. | g5178 | ATCACCTGCACTACTCCTGG | GCCGAGAGATCCTGATGTGA | 245 |
| 6. | g5526 | CAATTGTCCTAGCCTTCCTGG | TCAAGTAGATAAAGTCACCAGCC | 147 |
| 7. | g9483 | GACGATGTTCTCCCAGTCATG | TCCAACCTGTCACCGATTTC | 299 |
| 8. | g9489 | GACGATGTTCTCCCAGTCATG | TCCAACCTGTCACCGATTTC | 299 |
| 9. | g9762 | GCCCCAAAACCATCAGAAC | CCTCTGCATGAACCCACTATG | 351 |
| 10. | g11810 | TTCGAGACTCAATGGCATCC | AGTTCAAGGCCAAGGGTAAG | 147 |
| 11. | g12149 | TTAGCTGCGCCCAGATTAGT | TAACAGCTTGCGGGAGAGTT | 241 |
| 12. | g12587 | GGTAAGAACGATGCAGACACAG | AAGGACGGTTGAGAATAGCAG | 139 |
| 13. | g12667 | ACTGCGGAACCTACAACCAC | ACGACAGGGCTGATGTAACC | 197 |
| 14. | g12698 | TCAACATCAACGCCACTACC | ACACAGTCCACACCAATATCC | 247 |
| 15. | g13285 | TGCTTTGACCTTCACCACTG | CAACCGTCCTTGAGAAGCTC | 234 |
| 16. | g13528 | GCTATTTCCCGCCTTTTGTG | GACTACCTTCCACTGTCCATG | 228 |
